# Docking rings in a solid: reversible assembling of pseudorotaxanes inside a zirconium metal–organic framework†

**DOI:** 10.1039/d2sc01497a

**Published:** 2022-05-02

**Authors:** Xia Li, Jialin Xie, Zhenglin Du, Long Jiang, Guangqin Li, Sanliang Ling, Kelong Zhu

**Affiliations:** School of Chemistry, Sun Yat-Sen University Guangzhou 510275 China zhukelong@mail.sysu.edu.cn; Instrumental Analysis and Research Centre, Sun Yat-Sen University Guangzhou 510275 China; Advanced Materials Research Group, Faculty of Engineering, University of Nottingham Nottingham NG7 2RD UK Sanliang.Ling@nottingham.ac.uk

## Abstract

An unprecedented zirconium metal–organic framework featuring a T-shaped benzimidazole strut was constructed and employed as a sponge-like material for selective absorption of macrocyclic guests. The neutral benzimidazole domain of the as-synthesized framework can be readily protonated and fully converted to benzimidazolium. Mechanical threading of [24]crown-8 ether wheels onto recognition sites to form pseudorotaxanes was evidenced by solution nuclear magnetic resonance, solid-state fluorescence, and infrared spectroscopy. Selective absorption of [24]crown-8 ether rather than its dibenzo counterpart was also observed. Further study reveals that this binding process is reversible and acid–base switchable. The success of docking macrocyclic guests in crystals *via* host–guest interactions provides an alternative route to complex functional materials with interpenetrated structures.

## Abstract 2

A T-shaped ligand was designed as struts for building a zirconium metal–organic framework. Acid–base switchable docking and releasing a 24-membered crown ether inside crystals was successfully accomplished *via* post-synthetic modification.

## Graphical abstract

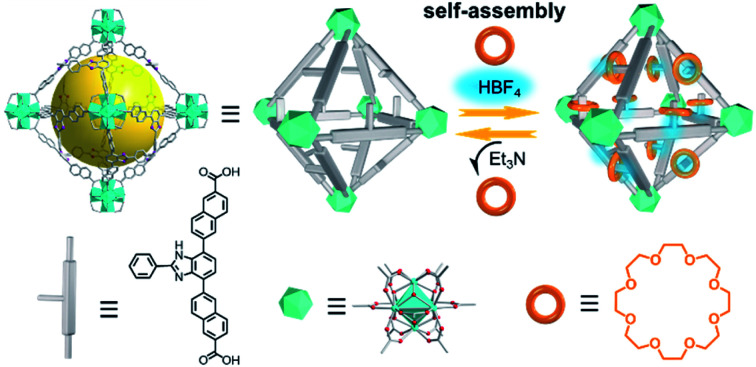

## Introduction

Rotaxanes and pseudorotaxanes are important interpenetrated molecular architectures consisting of macrocyclic wheels and axle molecules.^1^ The dynamic and switchable nature of their structures facilitates wide applications in the design of artificial molecular machines^2^ and advanced catalysts.^3^ Rotaxane-derived rotors,^4^ shuttles,^5^ pumps,^6^ transporters,^7^ and a peptide synthesizer^8^ have been successfully constructed to mimic the motions and functions of biological machines in Nature. Moreover, the reversible threading and de-threading of a pseudorotaxane enables advanced systems for drug delivery,^9^ fluorescence sensing,^10^ and self-healing polymers.^11^ Despite these wide-spread applications, organizing pseudorotaxanes or rotaxanes into crystal lattices to develop solid-state molecular machinery^12^ or stimuli-responsive materials^13^ is relatively less explored.

**Fig. 1:**
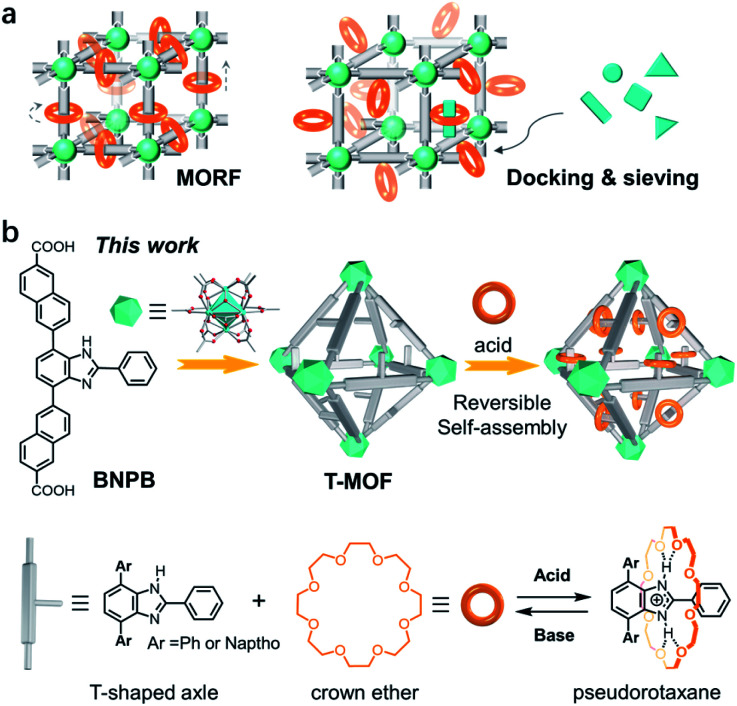
(a) Schematic display of metal–organic rotaxane frameworks (MORFs) and MOFs with active macrocyclic sites capable of docking and sieving guest molecules. (b) A zirconium MOF constructed with a T-shaped benzimidazole-derived ligand and its application in reversible self-assembly of pseudorotaxanes with crown ether wheels in the framework, *i.e*., docking rings in solids.

Metal–organic frameworks (MOFs), also known as porous coordination polymers (PCPs), are porous materials with periodic and tailorable structures.^14^ The high stability and large pore size have proven themselves ideal platforms for accommodating and operating bulky molecular assemblies.^15^ The incorporation of rotaxanes into MOFs affords metal–organic rotaxane frameworks (MORFs) which are potentially dynamic materials furnishing motions of molecular machines (Fig. 1a).^12*a*,16^ The most representative work has been achieved by Loeb and co-workers demonstrating that both rotational and translational motions can be unambiguously accessed in UWDM serial materials, paving the way to solid-state molecular machinery.^17^ In 2009, an outstanding example of assembling pseudorotaxanes inside MOFs was reported by Stoddart and co-workers.^18^ With macrocyclic recognition modules incorporated into a framework, zinc MOF-1001 is capable of docking and sieving dication paraquat guests (Fig. 1a). Although this process can be reversed by rinsing with solvents, efficiently docking and releasing a threaded component in a controlled fashion remains a great challenge.

The UiO series of zirconium-based MOFs are known for their robustness which facilitates post-synthetic modifications and switchable applications.^19^ A mechanized Zr MOF for photoresponsive cargo release was demonstrated by Wang *et al.*^13*a*^ More recently, Zr MOFs incorporated with benzimidazole rotaxane shuttles exhibit extraordinary acid–base stability which should enable the further design and installation of molecular switches in crystals.^20^ These inspiring studies have led us to propose a post-synthetic approach to form pseudorotaxanes in a pre-constructed framework *i.e*. docking rings in a solid (Fig. 1b). Specifically, a naphthalene-elongated T-shaped benzimidazole ligand was designed and synthesized to form a three-dimensional crystalline framework with zirconium (T-MOF, T represents thread).^21^ The robustness and large cavities of the material facilitate further acid–base doping modification. Upon protonation of the benzimidazole domains to benzimidazolium, crystal lattices with recognition sites were readily produced. Finally, reversible self-assembly of pseudorotaxanes with crown ether wheels in crystals was accomplished realizing the docking of rings in a solid.

## Results and discussion

**Scheme 1:**
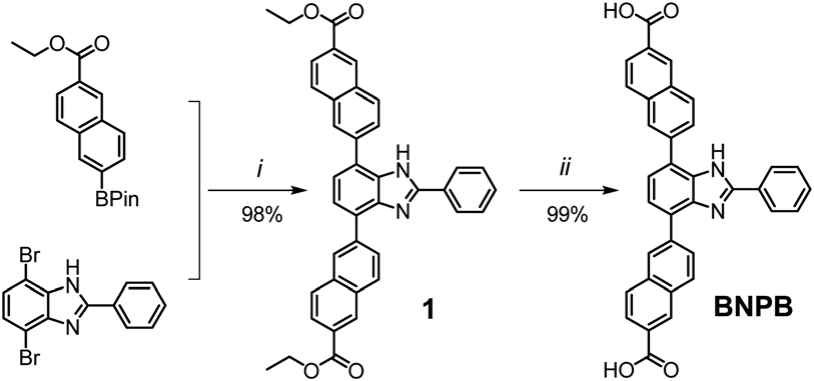
Synthesis of the T-shaped dicarboxylic acid ligand BNPB. Conditions: (i) Pd(Ph_3_P)_4_, Na_2_CO_3_, THF/H_2_O, reflux 24 h; (ii) 1 M NaOH reflux 24 h, then 1 M HCl.

**Fig. 2:**
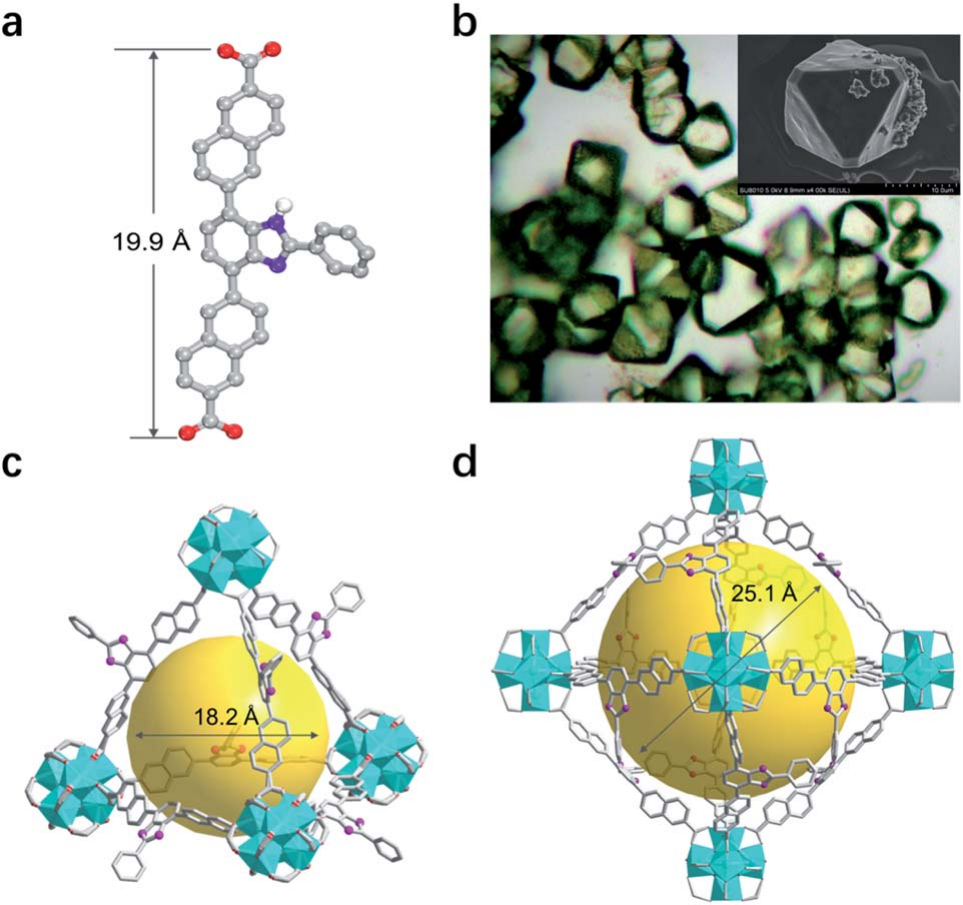
(a) Single-crystal structure of BNPB. (b) Optical and scanning electron microscopy images of T-MOF crystals. (c) The tetrahedral and (d) octahedral cavities in the single-crystal structure of T-MOF. (Color codes: C = gray; O = red; N = violet; Zr = cyan; H atoms are omitted for clarity).

Key steps for the synthesis of the T-shaped dicarboxylic acid ligand, 4,7-bis(6′-carboxynaphthalen-2′-yl)-2-phenyl-1*H*-benzimidazole (BNPB), are outlined in Scheme 1. The diester intermediate 1 was readily obtained by Pd-catalyzed Suzuki coupling of 4,7-dibromo-2-phenyl-1*H*-benzimidazole with 6-ethoxycarbonylnaphthalene-2-boronic acid pinacol ester. Hydrolysis of 1 afforded BNPB in excellent yield (99%). The structure of BNPB was unambiguously confirmed by NMR, mass spectrometry and single crystal X-ray diffraction (SCXRD) analyses (see ESI, Table S1†).^22^ The structure of BNPB shows that the two naphthalene wings are almost co-planar to the central benzene ring due to conjugation (Fig. 2a). The anti-co-conformation of two naphthalene wings results in a distance of 19.9 Å (*d*_O⋯O_) between the two carboxyl groups which is fairly close to that of the ligand reported for UiO-69.^23^ Accordingly, either an interpenetrated or non-interpenetrated framework could be obtained when combining BNPB with the 12-connection Zr_6_(μ_3_-O)_4_(μ_3_-OH)_4_ secondary building unit (SBU).

Upon heating BNPB and ZrCl_4_ in DMF with trifluoroacetic acid as the modulator at 100 °C for 5 days, octahedral crystals suitable for single-crystal X-ray diffraction were harvested and designated as T-MOF (Fig. 2b). The similar morphology of T-MOF to many other Zr-MOFs constructed with linear dicarboxylic linkers indicates that it is isoreticular to UiO-66.^19^ Despite the good crystal quality, T-MOF showed weak diffraction due to the porous nature and its inherent disorder in the framework. SCXRD analysis reveals that T-MOF crystallizes in the cubic space group *Fm*3̄ with a lattice parameter *a* = 38.49 Å (Table S1†), which is slightly larger than that reported for UiO-69.^23^ The Zr_6_(μ_3_-O)_4_(μ_3_-OH)_4_ SBUs are linked by BNPB to afford the 12-connected 3D network of fcu topology. It should be noted that, with the central benzimidazole moiety perpendicular to the struct, a non-interpenetrated framework is obtained exclusively with a variety of synthetic conditions. As depicted in Fig. 2c and d, two types of cages are found in the structure. The cavity sizes are estimated to be *ca.* 18.2 Å and 25.1 Å (in diameter) for the tetrahedron and octahedron, respectively. The central benzimidazole moiety is disordered over four positions and located in either of the two different cavities. The total solvent accessible volume of T-MOF is estimated to be 57.2% by analysis using the Olex2 (ref. 24) solvent mask (Olex2 implementation of the Platon Squeeze routine^25^). The large void implies potential application of T-MOF in accommodating large guest molecules.

To prove the phase purity of T-MOF as well as the integrity of the framework after solvent exchange, the powder X-ray diffraction (PXRD) patterns of the as-synthesized and dichloromethane (DCM) soaked phases were compared with the pattern calculated from the single crystal data (Fig. S2†). Slight shifting (0.9° to higher angles) was observed for as-synthesized T-MOF measured at 298 K. The discrepancy can be attributed to the different temperatures used to determine the PXRD of bulk and single crystals. Accordingly, Rietveld refinement of the cell parameter was further applied (Fig. S3†). The refined lattice parameter (*a*′ = 38.35 Å) afforded a simulated pattern in good consistence with those of as-synthesized and DCM soaked materials. Thermal gravimetric analysis (TGA) revealed that activated T-MOF starts to undergo slow degradation above 140 °C indicating a lower thermal stability compared to those reported for Zr MOF analogues (Fig. S4†). This is presumably due to the non-interpenetrated framework with a long strut. N_2_ sorption isotherms at 77 K of the activated T-MOF show a Brunauer–Emmett–Teller (BET) surface area of 200 m^2^ g^−1^ (Fig. S5†), a much smaller number than its theoretical BET surface area (Fig. S22†), suggesting partially impaired porosity upon removal of the solvents. Nevertheless, pseudorotaxanes are usually formed at ambient temperature in the presence of a solvent. Under such mild operating conditions, the stability of bulk T-MOF crystals should be sufficient to ensure the self-assembling process.

T-shaped benzimidazoliums have been proven to efficiently form pseudorotaxanes with 24-membered crown ethers.^21^ To probe the ability of T-MOF to bind crown ethers, a model compound [1-H][BF_4_] was first tested for its host–guest chemistry with [24]crown-8 (24C8) and dibenzo[24]crown-8 (DB24C8) ethers. The association constants *K*_a_ for complexes [1-H⊂24C8][BF_4_] and [1-H⊂DB24C8][BF_4_] in CD_3_CN were determined to be 793 and 1097 M^−1^, respectively (Fig. S6–9†). The higher binding affinity for DB24C8 to [1-H]^+^ could be attributed to π-stacking interactions resulting from the clamping of the crown around the axle as previously reported.^21^ In addition, disassembling both pseudorotaxanes was readily realized by neutralization with a base.

**Fig. 3:**
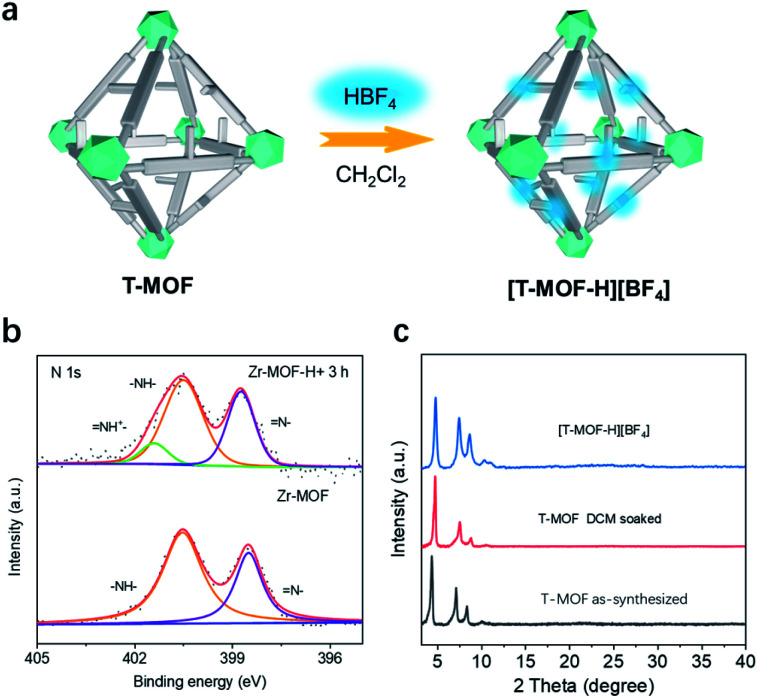
(a) Cartoon representation of protonating T-MOF to [T-MOF-H][BF_4_]. (b) X-ray photoelectron spectroscopy (XPS) comparison of T-MOF with protonated T-MOF. (c) PXRD patterns of as-synthesized, DCM soaked T-MOF, and [T-MOF-H][BF_4_].

Accordingly, the neutral as-synthesized T-MOF was subjected to protonation (Fig. 3a). To obtain the protonated material [T-MOF-H]^+^, CH_2_Cl_2_ pre-exchanged T-MOF was soaked in a 0.01 M CH_2_Cl_2_ solution of HBF_4_ and continuously monitored by X-ray photoelectron spectroscopy (XPS) analysis (Fig. 3b and Table S2†). The N 1s spectrum of the neutral T-MOF exhibits peaks at ∼398.5 and ∼400.5 eV which can be attributed to the imine (=N–) and amine (–NH–) moieties of the imidazole rings, respectively. Upon protonation, a new band at ∼401.5 eV for (–NH^+^=) appears (Fig. 3b) and corresponds to the signal observed for [1-H][BF_4_] (Fig. S20c and S20d†). This process was also evidenced by the fluorescence emission change upon protonation. Both the neutral ligand BNPB and T-MOF emit blue light with *λ*_max_ values of 439 and 435 nm respectively, while the band for [T-MOF-H][BF_4_] is centered at 474 nm when excited at 365 nm (Fig. S11 and 12†). The conversion efficiency was determined by the atomic ratio of Zr/B observed on XPS (Table S2†). Full protonation was achieved after soaking for 3 h at room temperature. The completely protonated T-MOF crystal retains its octahedral shape but some surface cracks are observed (Fig. S13†). Although peak broadening was detected in the PXRD pattern (Fig. 3c) which implies some loss of crystallinity, this did not significantly impede the study of pseudorotaxane formation in the protonated crystals.

**Fig. 4:**
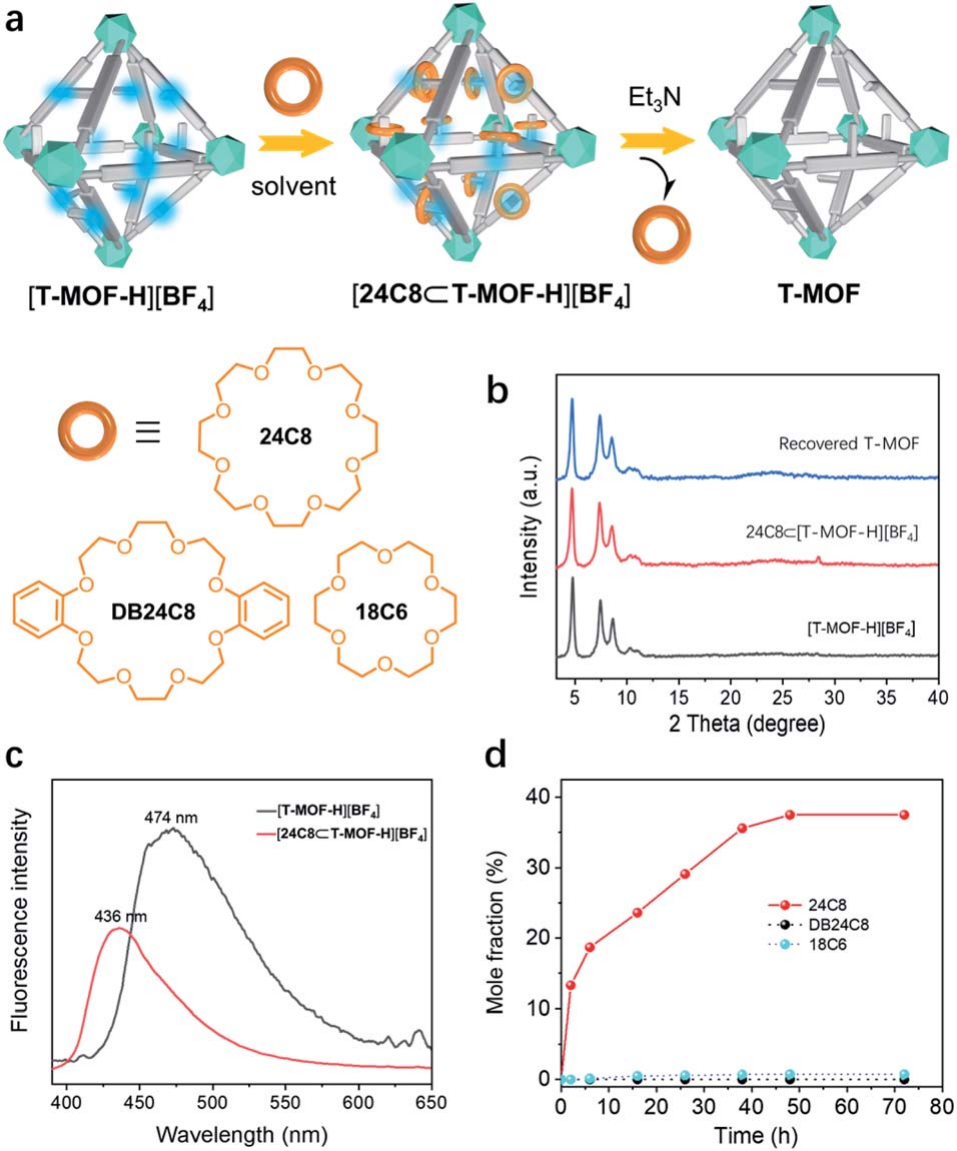
(a) The acid–base switchable uptake/release of various crown ethers inside the T-MOF. (b) PXRD patterns of [T-MOF-H][BF_4_], [24C8⊂T-MOF-H][BF_4_], and recovered T-MOF. (c) Comparison of the solid-state fluorescence emission of [T-MOF-H][BF_4_] with [24C8⊂T-MOF-H][BF_4_]. *E*_x_ = 365 nm. (d) Molar fraction plot of crown ether/[T-MOF-H][BF_4_] *versus* time.

With [T-MOF-H][BF_4_] in hand, ring docking experiments were carried out utilizing 24C8, DB24C8, and 18C6 (Fig. 4a). Immersion of freshly prepared [T-MOF-H][BF_4_] in a solution of 24C8 in CH_2_Cl_2_ (100 mg mL^−1^) for 2 days afforded a solid [24C8⊂T-MOF-H][BF_4_] with retained crystallinity (Fig. 4b). Its decreased and blue-shifted fluorescence emission at 436 nm (Fig. 4c) indicated host–guest interactions between [T-MOF-H][BF_4_] and 24C8.^26^ This was corroborated by infrared spectroscopy (IR) of the soaked material as shifting of the N–H stretching band from 3401 to 3214 cm^−1^ infers the formation of N–H⋯O hydrogen bonding (Fig. S21†). Further analysis of a digested sample (K_3_PO_4_/D_2_O/DMSO-*d*_6_) by ^1^H NMR spectroscopy gave a molar ratio of *ca.* 0.4 for wheels to benzimidazolium domains (Fig. 4d and S14†). Conversely, performing the same docking experiments with 18C6, which is known to be too small to thread a phenyl group (Fig. S17a†),^27^ resulted in no change in either the fluorescence or IR spectrum, and the absence of a crown ether signal in the ^1^H NMR (Fig. S17b†). This result rules out the possibility that absorption of the 24C8 of rings is due to non-specific electrostatic interactions or adherence to the crystal surface, proving that docking of 24C8 in the protonated MOF is dictated by mechanical bonding associated with the formation of pseudorotaxanes. As outlined in Fig. 4d, fast loading of 24C8 was achieved in the first 6 h (18.7%) and the molar fraction increased to 36% after immersion for 40 h. A maximum of 37.5% (*ca.* 0.4 wheel per benzimidazolium domain) was reached in 48 h. No significant change was observed for prolonged processing indicating the saturation of binding. T-MOF consists of two types of cavities, a large octahedral cage and a smaller tetrahedral cage. Each octahedral cage is face sharing with 8 tetrahedral cages, that gives a ratio of 2 : 1 for the tetrahedron/octahedron in the crystal. Since the volume of a regular octahedron is four times that of a regular tetrahedron, the statistical distribution ratio of “side arms” in cavities should be 1 : 2 for the tetrahedron/octahedron. Considering that the smaller tetrahedral cavity is likely to exhibit a lower binding energy due to the steric restriction of the smaller pore,^20^ the ring/linker ratio of 0.4 presumably means that most of the rings are trapped in the octahedral cages to form pseudrotaxanes. To our surprise, attempts to construct a similar material utilizing DB24C8 as the wheel component result in no absorption (Fig. 4d and S16†). This could be accounted for by the bulky size and rigidity of DB24C8 as compared to 24C8. Based on reported [2]pseudorotaxane structures, DB24C8 usually adopts the C-shaped clamped conformation when it is threading on a benzimidazolium axle.^21^ An estimated dimension of *ca.* 13.8 Å is about the smallest size for a complexed DB24C8 (Fig. S24†), while it is bigger than the predominant pores with diameters ranging from 10.0 to 12.6 Å according to the calculated pore size histogram (Fig. S23†).

Finally, the reversibility of the docking process was evaluated. As previously noted, disassembly of benzimidazolium pseudorotaxanes can be readily realized by neutralization with a base. To verify this, a ring (24C8) threading experiment was first conducted on neutral T-MOF with no detectable signal of crown ether observed by ^1^H NMR (Fig. S15†). A ring de-threading experiment was then executed by suspending [24C8⊂T-MOF-H][BF_4_] in a triethylamine/CH_2_Cl_2_ solution (0.01 mol mL^−1^). ^1^H NMR analysis revealed that disassembling of pseudorotaxane inside the solid was rapid and completed within 30 min (Fig. S19†). The recovery of T-MOF, as confirmed by the PXRD pattern (Fig. 4b), further proves that reversible assembly of pseudorotaxanes inside a metal–organic framework, *i.e*. docking rings in solids, can be achieved.

## Conclusions

To conclude, a novel T-shaped benzimidazole ligand was designed and utilized as struts for the preparation of a zirconium-based metal–organic framework. The neutral domains of the as-synthesized framework could be readily converted to recognition sites for templating pseudorotaxanes with appropriately sized crown ethers. The resulting sponge-like material exhibits selective and acid–base switchable absorption of 24C8 while rebuffing its dibenzo counterpart DB24C8 or the smaller 18C6. The success of reversibly docking and releasing rings inside a crystalline solid provides further impetus for exploration into related phenomena such as mechanisorption.^15*d*^

## Experimental

### Synthesis of BNPB

A 250 mL round-bottom flask was charged with compound 1 (0.295 g, 0.5 mmol, 1.0 equiv.), 1 M NaOH (15 mL, 15 mmol, 30 equiv.), tetrahydrofuran (20 mL) and ethanol (20 mL). After the reaction mixture was stirred at 80 °C for 12 h and TLC indicated the complete disappearance of the material, the solvent was removed in a vacuum. Deionized water was added to dissolve the residue and the solution was acidified with 1 M HCl to adjust the pH to 4–5, and the mixture was stirred for another 10 h. The precipitate was collected by vacuum filtration, washed with deionized water and air dried. Pale yellow solid ligand BNPB was obtained in 99% yield (0.265 g). ^1^H NMR (400 MHz, DMSO-*d*_6_) *δ* 13.16 (s, 2H), 12.89 (s, 1H), 8.90 (s, 1H), 8.72 (s, 1H), 8.67 (s, 1H), 8.53 (d, *J* = 8.4 Hz, 1H), 8.40 (s, 1H), 8.37–8.33 (m, 2H), 8.31 (d, *J* = 8.4 Hz, 1H), 8.26 (d, *J* = 8.8 Hz, 1H), 8.15 (d, *J* = 8.4 Hz, 2H), 8.06 (d, *J* = 10.8 Hz, 1H), 8.03 (d, *J* = 9.6 Hz, 1H), 7.97 (d, *J* = 8.4 Hz, 1H), 7.78 (d, *J* = 8.0 Hz, 1H), 7.58–7.48 (m, 4H). ^13^C NMR (100 MHz, DMSO-*d*_6_) *δ* 168.1, 153.5, 142.9, 138.6, 138.0, 135.7, 134.5, 132.1, 131.9, 130.9, 130.7, 130.6, 130.2, 129.8, 129.5, 129.3, 129.2, 128.9, 128.6, 128.2, 127.9, 126.2, 126.0, 124.2, 122.4. HRMS (APCI) calcd for C_35_H_23_N_2_O_4_^+^ [M + H]^+^ 535.1652 found: 535.1644.

### Synthesis of T-MOF

ZrCl_4_ (9.4 mg) and BNPB (10.7 mg) were dissolved in 2 mL of DMF in a 15 mL pressure tube, and 0.05 mL of trifluoroacetic acid was added. The mixture was then heated at 100 °C for 5 days, obtaining light yellow crystals (yield: 9.7 mg). The obtained crystals were immersed in dichloromethane with replacement of solvent very 12 h for 2 days prior to further characterization. Elemental Analysis (evacuated): C_210_H_128_N_12_O_32_Zr_6_, calcd: C, 65.03%; H, 3.33%; N, 4.33%; found: C, 63.04%; H, 3.57%; N, 4.11%.

## Data availability

All experimental and computational data associated with this article are included in the main text and ESI.†

## Author contributions

K. Z. supervised the project. X. L. performed all the synthetic experiments. X. L. collected and analyzed the NMR, PXRD, TGA, FT-IR, and XPS data with assistance from K. Z., J. X., Z. D., and G. Li. L. J. and K. Z. collected and analyzed the SCXRD data. S. L. supervised theoretical computation study, analysis and interpretation. K. Z. wrote the manuscript with input from X. L. and S. L.

## Conflicts of interest

There are no conflicts to declare.

## Supplementary Material

SC-013-D2SC01497A-s001

SC-013-D2SC01497A-s002
