# Supplementary material for: Docking rings in a solid: reversible assembling of pseudorotaxanes inside a zirconium metal–organic framework
Source: Chem Sci. 2022 May 2;13(21):6291–6. doi: 10.1039/d2sc01497a (PMC9159108; doi:10.1039/d2sc01497a)
Supplement: SC-013-D2SC01497A-s001 [file SC-013-D2SC01497A-s001.pdf]

Electronic Supplementary Material (ESI) for Chemical Science.
This journal is © The Royal Society of Chemistry 2022

Supporting Information

Docking Rings in Solids: Reversible Assembling of Pseudorotaxanes inside a Zirconium Metal–Organic Framework

Xia Li,[a] Jialin Xie,[a] Zhenglin Du,[a] Long Jiang,[b] Guangqin Li,[a] Sanliang Ling,*,[c] and Kelong Zhu*,[a]

[a] School of Chemistry, Sun Yat-Sen University, Guangzhou, 510275, China. E-mail: zhukelong@mail.sysu.edu.cn

[b] Instrumental Analysis and Research Centre, Sun Yat-Sen University, Guangzhou, 510275, China.

[c] Advanced Materials Research Group, Faculty of Engineering, University of Nottingham, Nottingham, NG7 2RD, United Kingdom. E-mail: Sanliang.Ling@nottingham.ac.uk

Table of Contents

		Page
**1**.	Materials and general methods	S2
**2.**	Synthesis of ligand **BNPB**	S3–S5
**3.**	Synthesis and characterization of **T-MOF**	S5–S8
**4.**	X-ray crystallography of **BNPB** and **T-MOF**	S9–S13
**5**	Host-guest chemistry of [**1-**H**]**[HB_4] with crown ethers	S14–S17
**6.**	Synthesis and characterization of [**T-MOF-H]**[BF_4]	S18–S20
**7.**	Assembling pseudorotaxanes inside **[T-MOF-H]**[BF_4]	S21–S26
**8.**	Computational modelling and simulated N_2 isotherm	S27–S29
**9.**	NMR spectra of Compounds	S30–S33
	Reference:	S33

1. Materials and General Methods

4,7-dibromo-2-phenyl-1*H*-benzimidazole (**2**)[S1] and 6-bromo-2-naphthoate (**3**)[S2] were synthesized according to literature. All reagents were purchased from commercial suppliers and used without further purification unless stated otherwise. Flash column chromatography was performed over silica gel (200-300 mesh). NMR spectra were recorded on a JEOL 400YH instrument. NMR spectra were internally referenced to tetramethylsilane (^{1}H) or alternatively, to the residual proton solvent signal (^{13}C). All ^{13}C NMR spectra were recorded with complete proton decoupling. High-resolution mass spectra were measured on a Bruker AutoFlex Times TOF. The single crystal X-ray diffraction (SCXRD) data was collected on an Agilent Sapphire3 Gemini Ultra single crystal diffractometer using using a CuKα (λ = 1.54178 Å). Powder X-ray diffraction (PXRD) was tested on Rigaku smartlab with CuKα (λ = 1.540598 Å). Thermogravimetric analysis from 30 ~ 810 °C was performed on a DTA-60 Simultaneous DTG-TG Apparatus (Shimadzu) under nitrogen atmosphere. XPS were recorded on a VG Scientific ESCALAB 250 instrument. Gas sorption isotherms measurements were carried out on a Micromeritics 3Flex Version 5.00 instrument. Fluorescence spectra were recorded on an FLS980 instrument. SEM images were collected on a Hitachi SU8010 system.

2. Synthesis of ligand BNPB

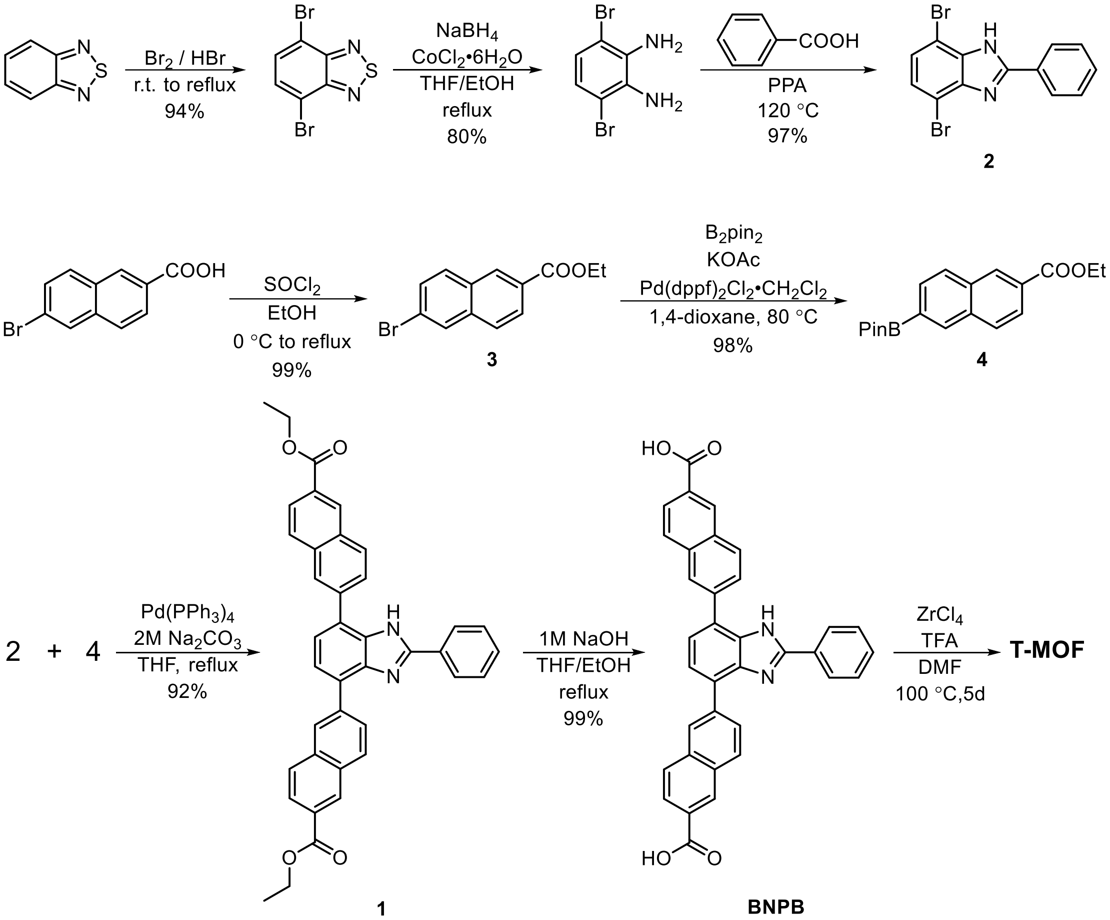

1 BNPB

Scheme 1. Synthetic steps involved in the preparation of **T-MOF**.

6-Ethoxycarbonylnaphthalene-2-boronic acid pinacol ester (4)

A 250 mL three-necked round-bottomed flask was charged with compound ethyl 6-bromo-2-naphthoate **3** (4.854g, 17.4 mmol, 1.0 equiv), bis(pinacolato)diboron (4.850 g, 19.1 mmol, 1.0 equiv), [1,1'- bis(diphenylphosphino)ferrocene]palladium(II) dichloride dichloromethane adduct (0.710 mg, 0.87 mmol, 0.05 equiv) and potassium acetate (5.123 g, 52.2 mmol, 3.0 equiv) in a solution of 1,4-dioxane (60 mL) under argon atmosphere was stirred and heated at 80 °C for 12 h. After cooling down to room temperature, the reaction mixture was filtered through Celite®, the solvent was removed under reduced pressure and the residue was purified by column chromatography on silica gel with (petroleum ether / ethyl acetate =7/1) as eluent to give the product **4** (5.562 g, 98% yield) as a yellow solid. ^{1}H NMR (400 MHz, Chloroform-*d*) δ 8.59 (s, 1H), 8.39 (s, 1H), 8.06 (dd, J = 8.4, 1.6 Hz, 1H), 7.98 – 7.85 (m, 3H), 4.44 (q, J = 7.2 Hz, 2H), 1.44 (t, J = 7.2 Hz, 3H), 1.39 (s, 12H). ^{13}C NMR (100 MHz, Chloroform-*d*) δ 166.8, 136.0, 134.9, 134.1, 131.2, 130.8, 128.9, 128.7, 128.4, 125.3, 84.2, 61.2, 25.0, 14.5. HRMS (APCI) calcd for $C_{19}H_{24}BO_4^+$ [M+H]$^+$ 327.1762, found:327.1763.

Compound 1: A 250 mL Schlenk flask was charged with compound **2** (1.056 g, 3.0 mmol, 1.0 equiv), compound **4** (2.936 g, 9.0 mmol, 3.0 equiv), tetrakis(triphenylphosphine)palladium (0.520 g, 0.45 mmol, 0.15 equiv), Na_2CO_3 (2M in H_2O, 22 ml) and THF (88 mL) under argon atmosphere. After heating at refluxing for 12 h, the reaction mixture was cooled down to room temperature and the organic solvent was removed under reduced pressure, then H_2O and ethyl acetate were added and then the organic layer was separated, dried with anhydrous Na_2SO_4, filtered and concentrated in vacuum. The crude product was purified by column chromatography on silica gel with (petroleum ether / DCM =1/4) as eluent to give the product **1** (1.630 g, 92% yield) as a pale yellow solid. ^{1}H NMR (400 MHz, Chloroform-*d*) δ 10.52 (s, 1H), 8.68 (s, 1H), 8.59 (s, 1H), 8.43 (s, 1H), 8.35 (dd, J = 8.8, 1.6 Hz, 1H), 8.22 – 8.18 (m, 2H), 8.07 (s, 1H), 8.05 (dd, J = 8.8, 1.6 Hz, 1H), 8.02 (d, J = 8.8 Hz, 1H), 7.97 – 7.91 (m, 3H), 7.81 – 7.76 (m, 2H), 7.63 (d, J = 7.6 Hz, 1H), 7.49 – 7.41 (m, 4H), 4.44 – 4.35 (m, 4H), 1.44 (td, J = 7.2, 4.2 Hz, 6H). ^{13}C NMR (100 MHz, Chloroform-*d*) δ 167.1, 166.9, 152.7, 142.7, 138.4, 138.3, 135.9, 135.7, 133.6, 131.9, 131.7, 131.2, 130.7, 130.33, 130.30, 130.0, 129.3, 129.0, 128.7, 128.4, 128.2, 128.2, 127.8, 127.6, 127.2, 127.1, 126.7, 125.9, 125.4, 124.9, 123.8,

122.8, 61.4, 61.2, 14.5. HRMS (APCI) calcd for $C_{39}H_{31}N_2O_4^+$ $[M+H]^+$ 591.2278, found:591.2282.

Compound [1-H][BF_4]: **1** (59.0 mg, 0.10 mmol, 1 equiv) was completely dissolved in CH_2Cl_2 (6 mL). Then tetrafluoroboric acid diethyl ether complex (21.0 mg, 0.13 mmol) was added in one portion, causing the precipitation of a solid. After allowed to stir for 1h, the resulting solid was filtered, washed with Et_2O and dried under vacuum. White solid **[1-H][BF_4]** was obtained in 99% yield (67.0 mg). ^{1}H NMR (400 MHz, CD_3CN) δ 12.46 (s, 1H), 8.78 (s, 2H), 8.38 (s, 2H), 8.32 (d, J = 8.4 Hz, 2H), 8.18 (s, 4H), 8.07 (d, J = 7.6 Hz, 2H), 7.95 (dd, J = 8.4, 2.0 Hz, 2H), 7.91 (s, 2H), 7.79 (t, J = 7.6 Hz, 1H), 7.70 (t, J = 7.6 Hz, 2H), 4.44 (q, J = 7.2 Hz, 4H), 1.44 (t, J = 7.2 Hz, 6H). HRMS (APCI) calcd for $C_{39}H_{31}N_2O_4^+$ $[M - BF4]^+$ 591.2278 found:591.2282.

4,7-bis(6'-carboxynaphthalen-2'-yl)-2-phenyl-1*H*-benzimidazole (BNPB):
A 250mL round-bottom flask was charged with compound **1** (0.295 g, 0.5 mmol, 1.0 equiv), 1M NaOH (15 mL, 15 mmol, 30 equiv), tetrahydrofuran (20 mL) and ethanol (20 mL). After the reaction mixture was stirred at 80 °C for 12 h and TLC indicated the complete disappearance of the material, the solvent was removed in vacuum. Deionized water was added to dissolve the residue and acidified with 1M HCl to adjust pH 4–5, and the mixture was stirred for another 10 h. The precipitate was collected by vacuum filtration, wash with deionized water and air dried. Pale yellow solid ligand **BNPB** was obtained in 99% yield (0.265 mg). ^{1}H NMR (400 MHz, DMSO-d_6) δ 13.16 (s, 2H), 12.89 (s, 1H), 8.90 (s, 1H), 8.72 (s, 1H), 8.67 (s, 1H), 8.53 (d, J = 8.4 Hz, 1H), 8.40 (s, 1H), 8.37 – 8.33 (m, 2H), 8.31 (d, J = 8.4 Hz, 1H), 8.26 (d, J = 8.8 Hz, 1H), 8.15 (d, J = 8.4 Hz, 2H), 8.06 (d, J = 10.8 Hz, 1H), 8.03 (d, J = 9.6 Hz, 1H), 7.97 (d, J = 8.5 Hz, 1H), 7.78 (d, J = 8.0 Hz, 1H), 7.58 – 7.48 (m, 4H). ^{13}C NMR (100 MHz, DMSO-d_6) δ 168.1, 153.5, 142.9, 138.6, 138.0, 135.7, 134.5, 132.1, 131.9, 130.9, 130.7, 130.6, 130.2, 129.8, 129.5, 129.3, 129.2, 128.9, 128.6, 128.2, 127.9, 126.2, 126.0, 124.2, 122.4. HRMS (APCI) calcd for $C_{35}H_{23}N_2O_4^+$ $[M+H]^+$ 535.1652 found:535.1644.

3. Synthesis and characterization of T-MOF

$ZrCl_4$ (9.4 mg) and **BNPB** (10.7 mg) were dissolved in 2 mL of DMF in a 15 mL pressure

tube, and 0.05 mL of trifluoroacetic acid was added. The mixture was then heated at 100 ºC for 5 days, light yellow crystals were obtained (yield: 9.7 mg). Elemental analysis (evacuated): $C_{210}H_{128}N_{12}O_{32}Zr_6$, Calcd.: C, 65.03%; H, 3.33%; N, 4.33%; Found: C, 63.04%; H, 3.57%; N, 4.11%.

Activation (de-solvation) of **T-MOF**: The as-synthesized **T-MOF** was filtered and soaked in DMF for 24 hr. After the solvent was decanted, anhydrous (water≤30 ppm) CH_2Cl_2 was then added to the solids to exchange the residual DMF for 12 hr. Repeating the soaking process for another three times resulted in a CH_2Cl_2 exchanged material (**T-MOF** DCM soaked) ready for protonation or de-solvation. Prior to BET surface area measurement, the DCM soaked **T-MOF** was further exchanged once with dry hexanes. Subsequent evacuation at 100 °C for 24 hr afforded the desolated **T-MOF** which was kept in a desiccator before further use.

Digestion of **T-MOF**: In a typical procedure, saturated K_3PO_4/D_2O solution was well-mixed with DMSO-d_6 (V:V=2:1). Then, as-synthesized **T-MOF** samples were digested in the mixed solution and subjected to NMR measurements.

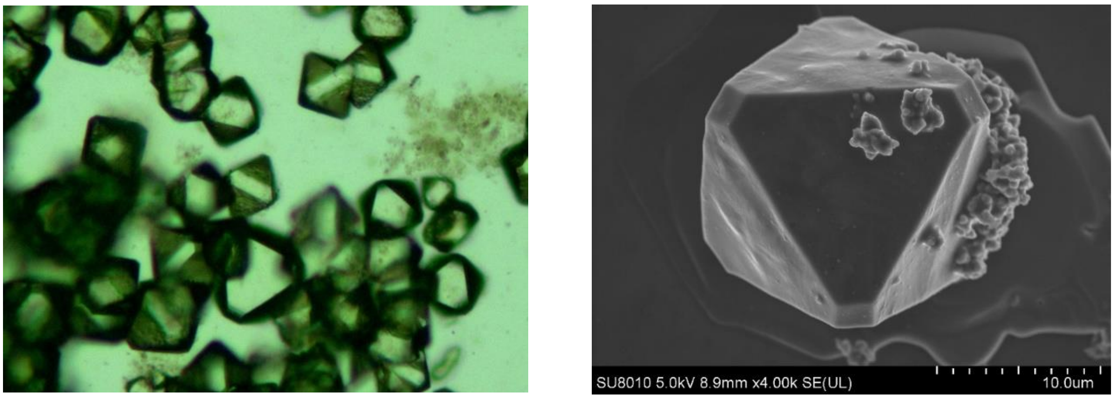

Figure S1. Optical (left) and SEM (right) images of as-synthesized **T-MOF**.

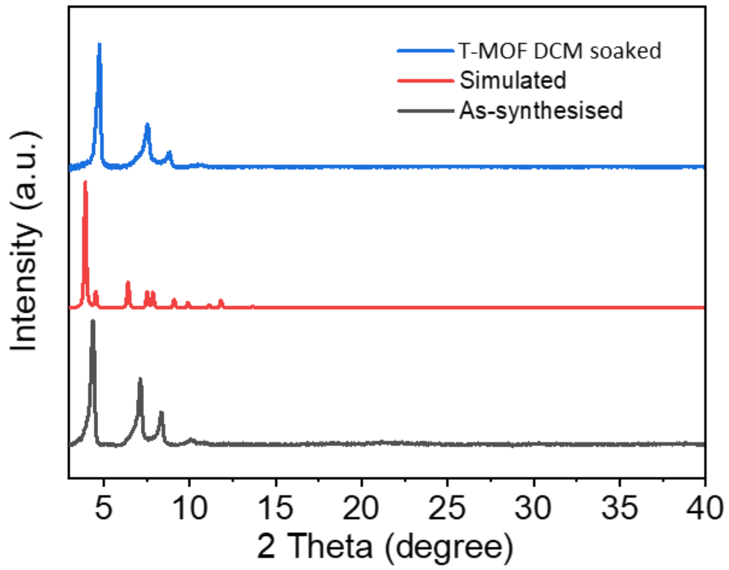

Figure S2. Powder X-ray diffraction pattern of as-synthesized **T-MOF** (black), simulated (red) and DCM soaked **T-MOF** (blue).

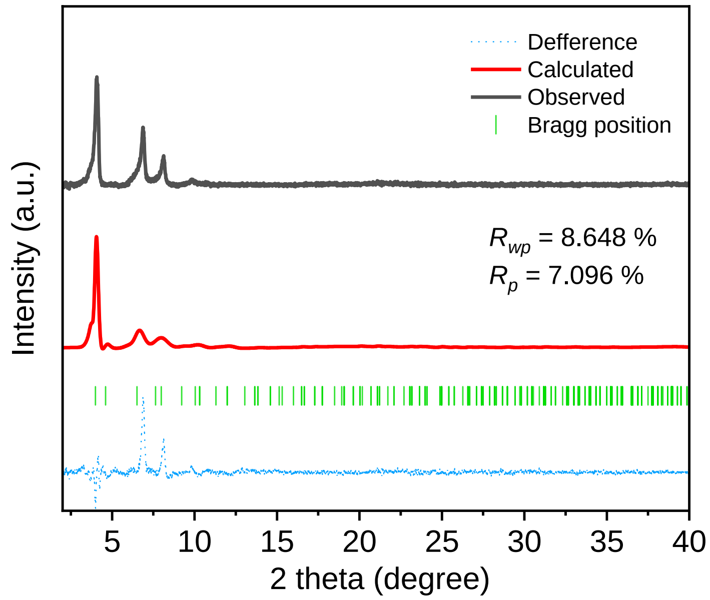

Figure S3. Rietveld refinement of the cell parameter for as-synthesized **T-MOF**. Lattice parameters: Crystal name: L3-2. **Cell parameters**: a=38.34565, b=38.34565, c=38.34565; alpha = 90.000, beta = 90.000, gamma = 90.000; Cell volume: 56383.01.

Final Rietveld parameters

Agreement factors

Rp = 7.096 Rwp = 8.648 Re = 99.671 Chi2= 0.008

Rp'= 42.972 Rwp'= 45.548 Re'= 524.934 DW= 1.186

R-structure factor = 12.296 R-Bragg factor = 21.335

Weighting scheme: w=1.0/ycount^2

Convergence criterium: eps=.000100

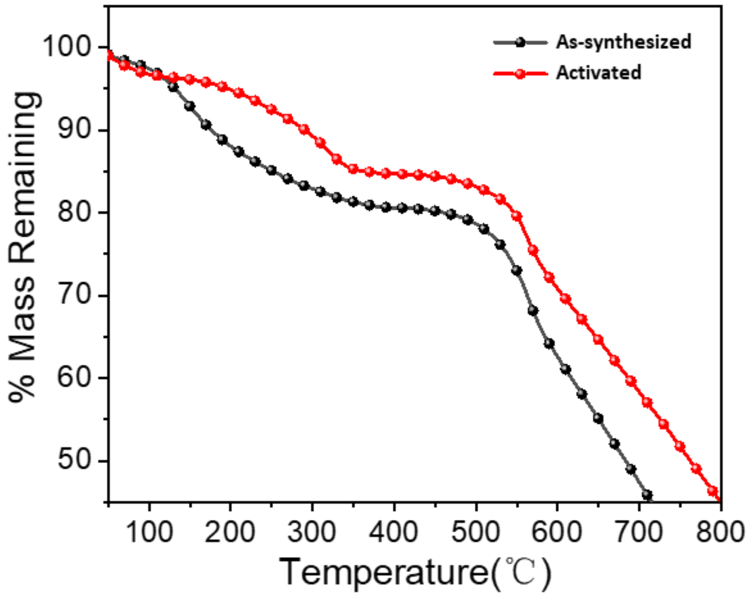

Figure S4. TGA analyses of as-synthesized (black) and activated (red) **T-MOF**.

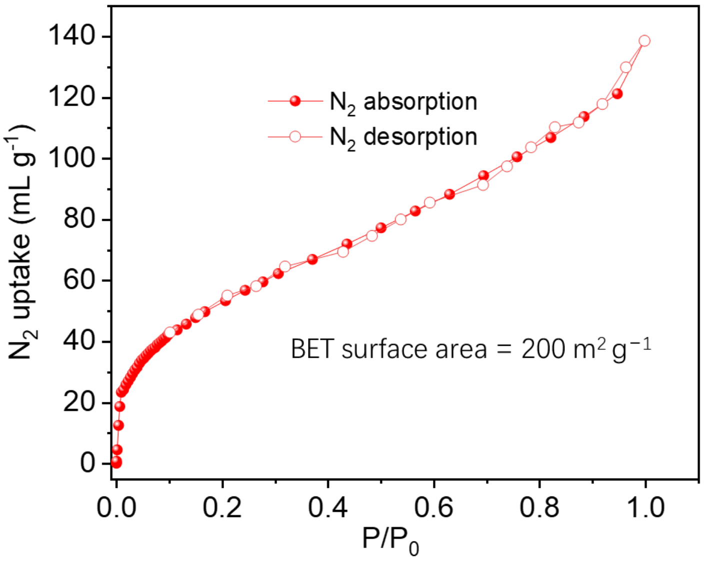

Figure S5. N_2 adsorption isotherms of activated **T-MOF** at 77 K.

4. X-Ray Crystallography of BNPB and T-MOF

Crystals were frozen in paratone oil inside a cryoloop under a cold stream of N_2. Reflection data were collected either on a Rigaku SuperNova, Dual, AtlasS2 diffractometer using monochromatized Cu Kα radiation or on a BRUKER D8 VENTURE PHOTON III diffractometer using Ga Kα radiation. Diffraction data and unit-cell parameters were consistent with assigned space groups. Lorentzian polarization corrections and empirical absorption corrections, based on redundant data at varying effective azimuthal angles, were applied to the data sets. The structures were solved using OLEX^2 crystallography software.[S3,S4] When practical, non-hydrogen atoms were refined anisotropically and hydrogen atoms placed in idealized positions and refined using a riding model. Figures were drawn with Diamond software. Details can be obtained from the Cambridge Crystallographic Data Centre at www.ccdc.cam.ac.uk for CCDC accession numbers 2120873 and 2120874.

Although high quality single crystals with suitable size were obtained for **T-MOF**, they showed very weak diffraction due to the porous nature and its inherent disorder in the framework. The central benzimidazole moiety between two naphthalene groups show rotational disorder around the strut could make the electron density very diffused. Nevertheless, positions of all the atoms in the SBUs (Zr, O, and carboxylate) and the struts could be unambiguously determined after several restraints were applied. The structure solution shows relatively high R values after refinement, and no further improvement was achieved after multiple data collection attempts on different crystals.

Refinement model description for T-MOF (restraints and constraints)

Details:

1. **Twinned data refinement**
  Scales: 0.528(14)
  0.472(14)
2. **Fixed Uiso**
  At 1.2 times of:
    All C(H) groups, All N(H) groups
  At 1.5 times of:
    All O(H) groups

3. **Restrained distances**

C3-C1 = C12-C10 = C19-C18 = C26-C15 = C35-C33
1.5 with sigma of 0.02
C1-O2 = C1-O3 = C35-O4 = C35-O5
1.25 with sigma of 0.02
N1-C17 = N1-C18 = N2-C18 = N2-C16
1.35 with sigma of 0.02
C16-C18 = C18-C17 = C17-N2 = N2-N1 = N1-C16
2.35 with sigma of 0.02
C1-C2 ≈ C1-C4 ≈ C10-C17 ≈ C10-C13 ≈ C12-C9 ≈ C12-C11 ≈
C18-C20 ≈ C18-C24 ~
C15-C25 ≈ C15-C27 ≈ C26-C14 ≈ C26-C16 ≈ C35-C32 ≈ C35-C34
with sigma of 0.02
O2-C3 ≈ O3-C3 ≈ O4-C33 ≈ O5-C33
with sigma of 0.01
N1-C19 ≈ N2-C19
with sigma of 0.01

4. **Restrained planarity**

C1, C2, C3, C4, C5, C6, C7, C8, C9, C10, C11, C12
with sigma of 0.1
C15, C25, C26, C27, C28, C29, C30, C31, C32, C33, C34, C35
with sigma of 0.1
O2, O3, C1, C3
with sigma of 0.01
O4, O5, C33, C35
with sigma of 0.01
N1, N2, C10, C12, C13, C14, C15, C16, C17, C18, C19, C26
with sigma of 0.1
C18, C19, C20, C21, C22, C23, C24
with sigma of 0.1

5. **Rigid bond restraints**

Zr1, O2
with sigma for 1-2 distances of 0.001 and sigma for 1-3 distances of 0.002

6. **Uiso/Uaniso restraints and constraints**

O2 ≈ O3 ≈ O4 ≈ O5 ≈ N1 ≈ N2 ≈ C1 ≈ C2 ≈ C3
≈ C4 ≈ C5 ≈ C6 ≈ C7 ≈ C8 ≈ C9 ≈ C10 ≈ C11 ≈
C12 ≈ C13 ≈ C14 ≈ C15 ≈ C16 ≈ C17 ≈ C18 ≈ C24 ≈
C19 ≈ C20 ≈ C21 ≈ C22 ≈ C23 ≈ C25 ≈ C26 ≈ C27 ≈
C28 ≈ C29 ≈ C30 ≈ C31 ≈ C32 ≈ C33 ≈ C34 ≈ C35:
within 2A with sigma of 0.04 and sigma for terminal atoms of 0.08 within 2A

7. **Others**

Fixed Sof: H1A(0.33333) O2(0.25) O3(0.25) O4(0.25) O5(0.25) N1(0.25)
H1(0.125) N2(0.25) H2(0.125) C1(0.25) C2(0.25) H2A(0.25) C3(0.25) C4(0.25)
H4(0.25) C5(0.25) H5(0.25) C6(0.25) C7(0.25) C8(0.25) H8(0.25) C9(0.25)

H9(0.25) C10(0.25) C11(0.25) H11(0.25) C12(0.25) C13(0.25) H13(0.25) C14(0.25) H14(0.25) C15(0.25) C16(0.25) C17(0.25) C18(0.25) C24(0.25) H24(0.25) C19(0.25) C20(0.25) H20(0.25) C21(0.25) H21(0.25) C22(0.25) H22(0.25) C23(0.25) H23(0.25) C25(0.25) H25(0.25) C26(0.25) C27(0.25) H27(0.25) C28(0.25) H28(0.25) C29(0.25) C30(0.25) C31(0.25) H31(0.25) C32(0.25) H32(0.25) C33(0.25) C34(0.25) H34(0.25) C35(0.25)

8.**a Riding coordinates**:

O1(H1A)

8.**b Aromatic/amide H refined with riding coordinates:**

N1(H1), N2(H2), C2(H2A), C4(H4), C5(H5), C8(H8), C9(H9), C11(H11), C13(H13), C14(H14), C24(H24), C20(H20), C21(H21), C22(H22), C23(H23), C25(H25), C27(H27), C28(H28), C31(H31), C32(H32), C34(H34)

8.**c Fitted hexagon refined as free rotating group:**

C12(C13,C14,C15,C16,C17), C24(C19,C20,C21,C22,C23)

8.**d Naphthalene refined as free rotating group:**

C2(C3,C4,C5,C6,C7,C8,C9,C10,C11), C25(C26,C27,C28,C29,C30,C31,C32,C33,C34)

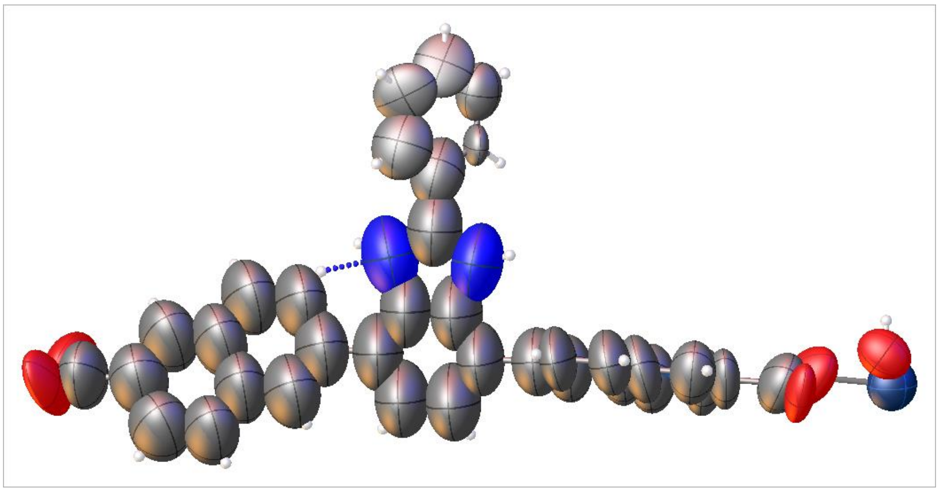

_refine_special_details: Refined as a 2-component twin.

_exptl_absorpt_process_details: CrysAlisPro 1.171.39.46 (Rigaku Oxford Diffraction, 2018) using spherical harmonicsas implemented in SCALE3 ABSPACK.

_platon_squeeze_special_details**:** A solvent mask was calculated and 3397 electrons were found in a volume of 31033Å^3 in 1 void per unit cell. This is consistent with the presence of 35[H_2O], 13[C_3H_7NO] per Formula Unit which account for 3480 electrons per unit cell. The solvent data was backed up by TGA result (Figure S4).

Comments on checkCIF A and B level alerts in structure of T-MOF:

Alert level A

```
THETM01_ALERT_3_A  The value of sine(theta_max)/wavelength is less than 0.550
            Calculated sin(theta_max)/wavelength =    0.4493
```

Author Response: Due to the low quality of the crystals data and the disorder of ligands, it was difficult to reach higher resolution using our machine for the structure.

Alert level B

```
PLAT084_ALERT_3_B High wR2 Value (i.e. > 0.25) ...................       0.37 Report
```

Author Response: This alert is caused by significant disorder in the structure.

```
PLAT088_ALERT_3_B Poor Data / Parameter Ratio ....................       6.16 Note
```

Author Response: This alert is caused by significant disorder in the structure.

```
PLAT260_ALERT_2_B Large Average Ueq of Residue Including       Zr1      0.344 Check
```

Author Response: This is due to the low quality of the crystals and disorder of ligands

Table S1. Crystal Data, Solution and Refinement Parameters.

	**BNPB**·$(C_2H_6SO)_2$	**T-MOF**
**CCDC number**	2120873	2120874
**formula**	$C_{39}H_{34}N_2O_6S_2$	$C_{249}H_{289}N_{25}O_{80}Zr_6$
**formula weight**	690.80	5459.35
**crystal system**	Triclinic	Cubic
**space group**	$P\bar{1}$	$Fm\bar{3}$
**T (K)**	150	150
**a (Å)**	9.9015(3)	38.4865(7)
**b (Å)**	11.8523(4)	38.4865(7)
**c (Å)**	14.5872(4)	38.4865(7)
**α (°)**	93.222(2)	90
**β (°)**	99.421(2)	90
**γ (°)**	100.578(1)	90
**V (Å³)**	1653.52(9)	57007(3)
**Z**	2	4
ρ, **g cm⁻³**	1.387	0.636
μ, **mm⁻¹**	1.226	1.214
**reflections used**	5454	1886
**variables**	493	306
**restraints**	45	432
R_1 $[I > 2\sigma(I)]$[a]	0.0667	0.1352
R_1 **(all data)**	0.0893	0.1457
$R_2w[I > 2\sigma(I)]$[b]	0.1626	0.3408
R_2w **(all data)**	0.1751	0.3661
**GoF on** F^2	1.048	1.679

[a] $R_1 = \Sigma ||F_o| - |F_c|| / \Sigma |F_o|$; [b] $R_2w = [\Sigma[w(F_o^2 - F_c^2)^2] / \Sigma[w(F_o^2)^2]]^{1/2}$, where $w = q[\sigma^2(F_o^2) + (aP)^2 + bP]^{-1}$

5. Host-guest chemistry of [1-H][BF_4] with crown ethers

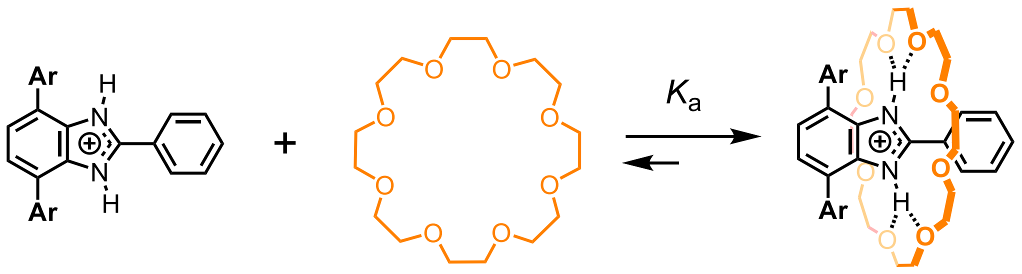

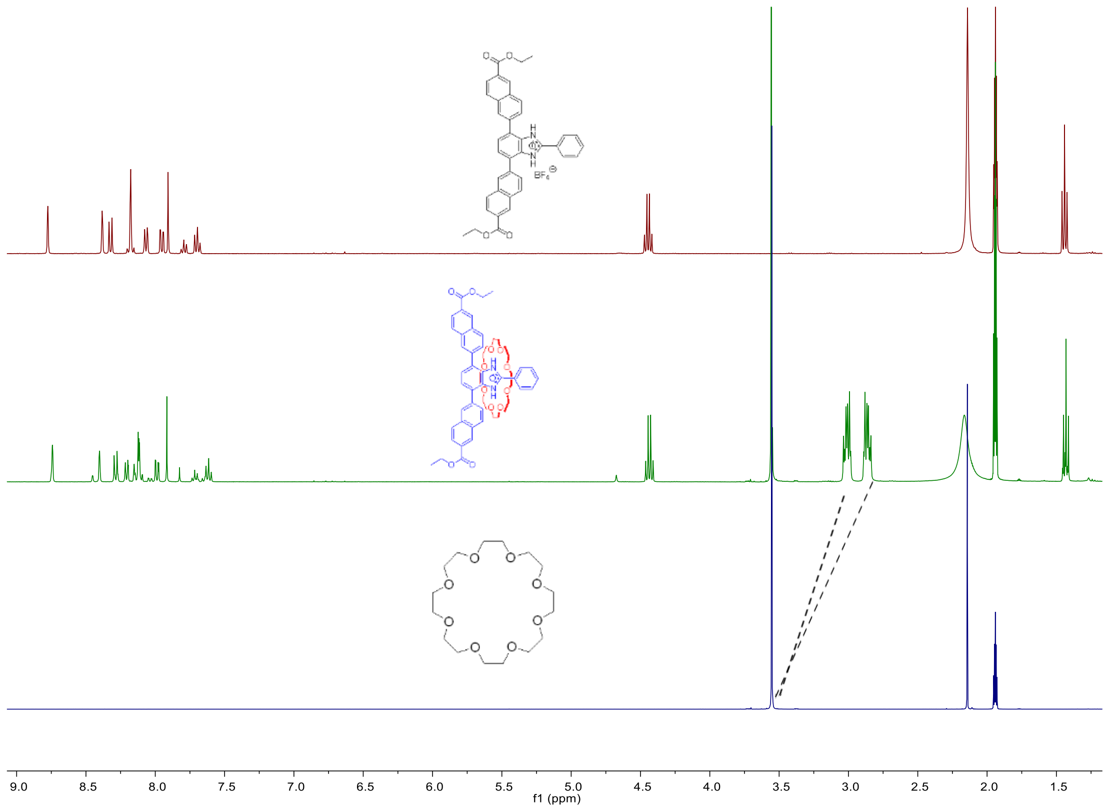

Figure S6. ^{1}H NMR spectra (400 MHz, 298 K, CD_3CN, 4.0 mM) of [**1**-H][BF_4] (top), equimolar mixture of [**1**-H][BF_4] and **24C8** (middle), and **24C8** (bottom).

The association constant (K_a) was calculated by a single-point method:

$$G + H \overset{K}{\rightleftharpoons} G\subset H \qquad K = \frac{[G\subset H]}{[G]\cdot [H]}$$

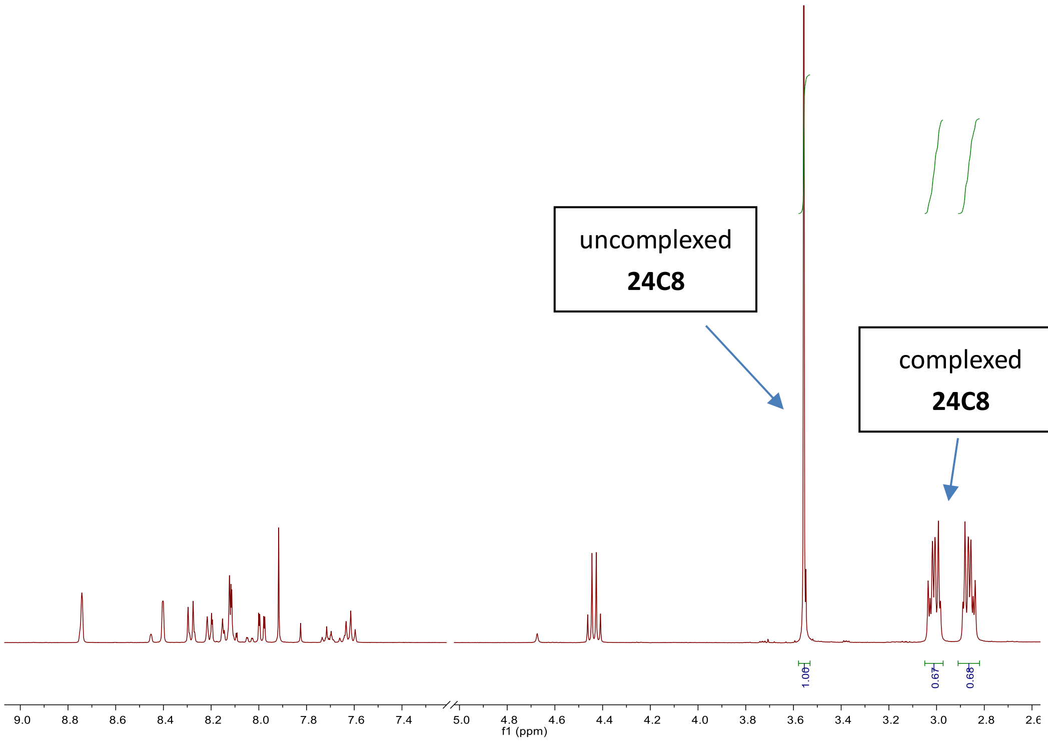

Figure S7. ^{1}H NMR spectrum (400 MHz, 298 K, CD_3CN 4.0 mM) of the equimolar mixture of [**1**-H][BF_4] and **24C8**.

$K_a = [(1.35/2.35) \times 4.0 \times 10^{-3}]/[(1.0/2.35) \times 4.0\times10^{-3}]/[(1.0/2.35)\times4.0\times10^{-3}] = 793\ M^{-1}$

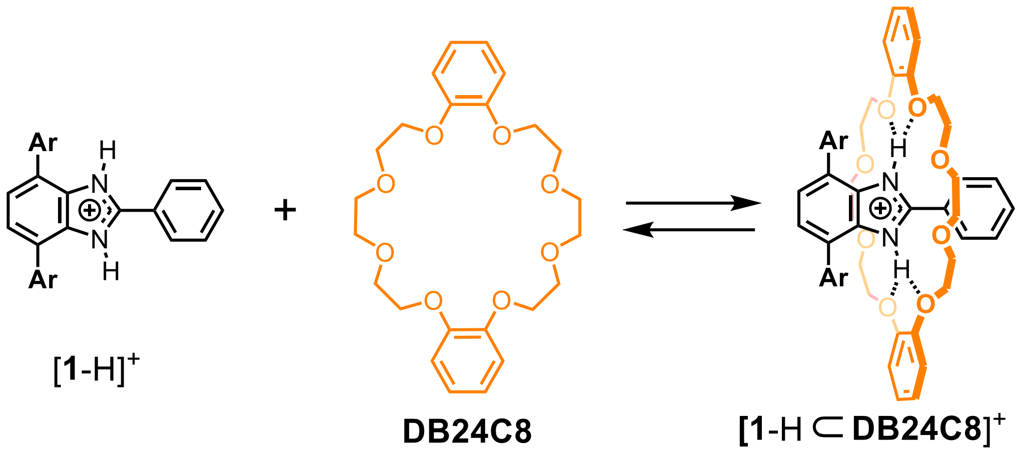

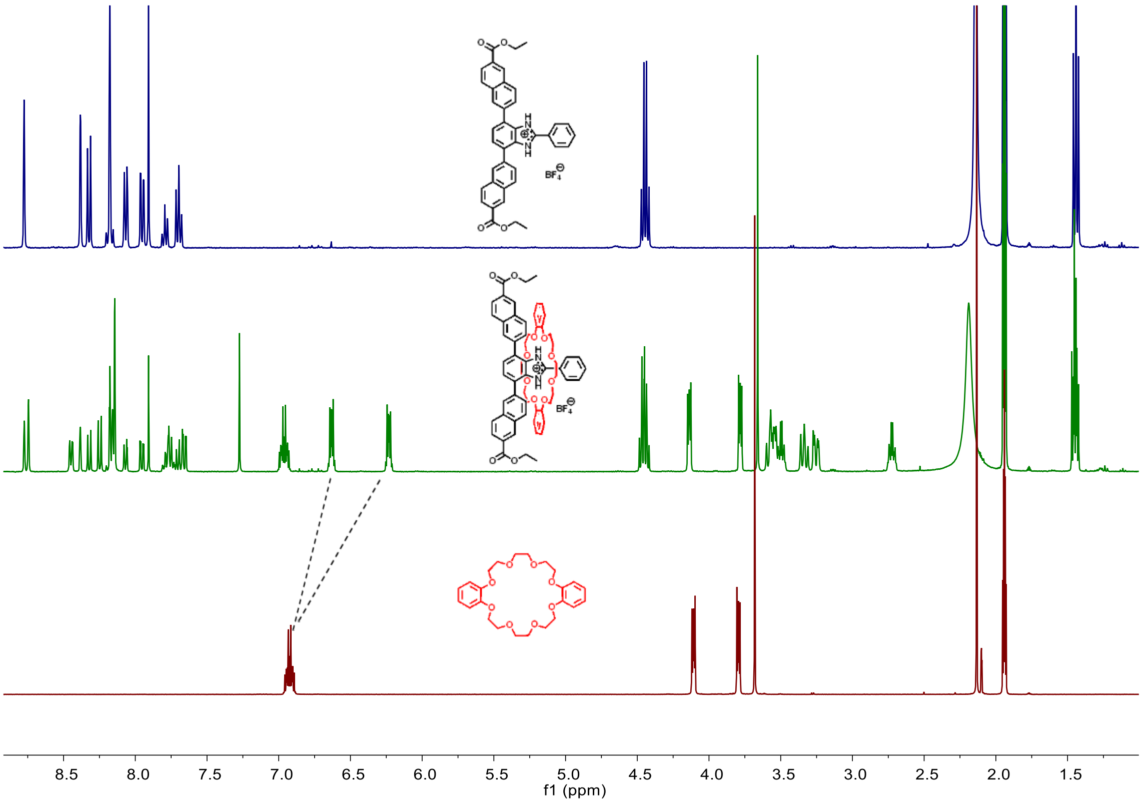

Figure S8. ^{1}H NMR spectra (400 MHz, 298 K, CD_3CN, 4.0 mM) of [**1**-H][BF_4] (top), equimolar mixture of [**1**-H][BF_4] and **DB24C8** (middle), and **DB24C8** (bottom).

The association constant (K_a) was calculated by a single-point method:

$$G + H \overset{K}{\rightleftharpoons} G\subset H \qquad K = \frac{[G\subset H]}{[G]\cdot[H]}$$

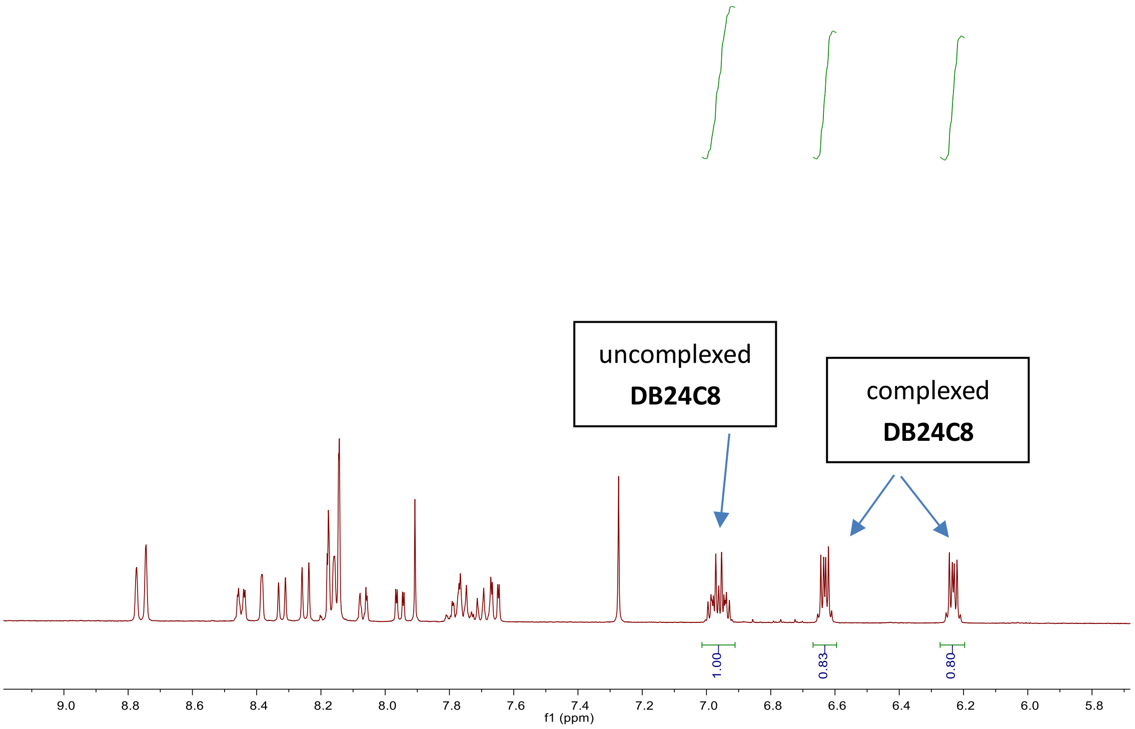

Figure S9. ^{1}H NMR spectrum (400 MHz, 298 K, $CDCl_3$ 4.0 mM) of the equimolar mixture of [**1**-H][BF_4] and **DB24C8**.

$$K_a = [(1.65/2.65)\times4.0\times10^{-3}]/[(1.0/2.65)\times4.0\times10^{-3}]/[(1.0/2.65)\times4.0\times10^{-3}] = 1097\ M^{-1}$$

6. Synthesis and characterization of [T-MOF-H][BF_4]

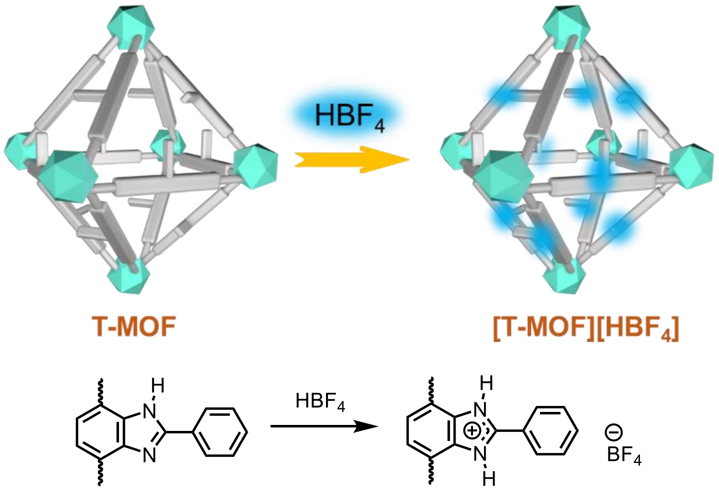

The protonated **T-MOF** was obtained by soaking the DCM exchanged **T-MOF** crystals in an acidic solution. In a typical procedure, 9.7 mg of as-synthesized **T-MOF** crystals were first immersed in super-dry (water⩽30 ppm) CH_2Cl_2 with replacing the solvent every 12 h for 2 days. After decanting the CH_2Cl_2, 1.0 mL of HBF_4 solution (0.01 M in super-dry CH_2Cl_2) was added. After soaking for a period (0~4 hours), the crystals were filtered, washed with CH_2Cl_2, and dried under N_2 atmosphere. The sample was kept in a desiccator for further studies.

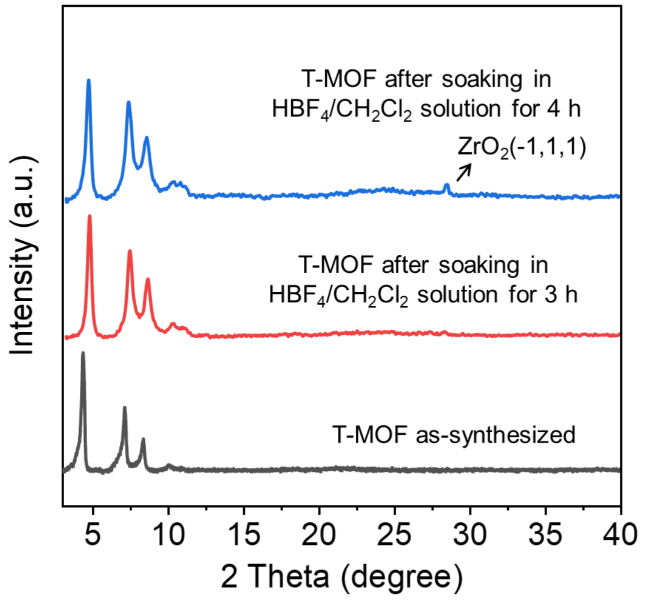

Figure S10. PXRD patterns of as-synthesized **T-MOF** and protonated **T-MOFs**.

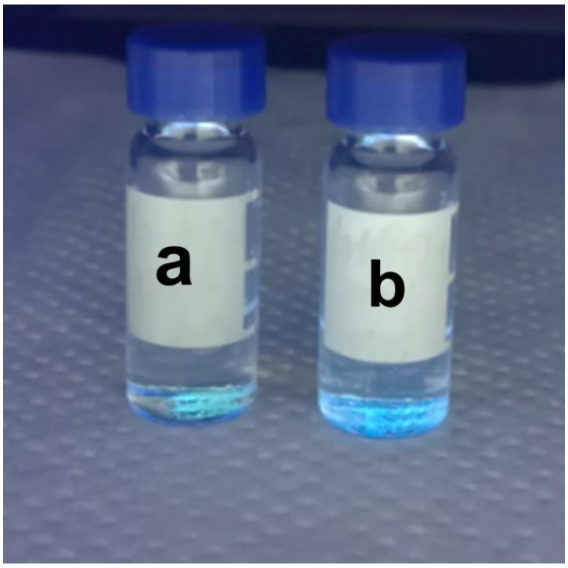

Figure S11. Images of neutral and protonated **T-MOFs**. (a) **T-MOF** DCM soaked. (b) **[T-MOF-H]**[BF_4]. Irradiated under UV lamp (365 nm).

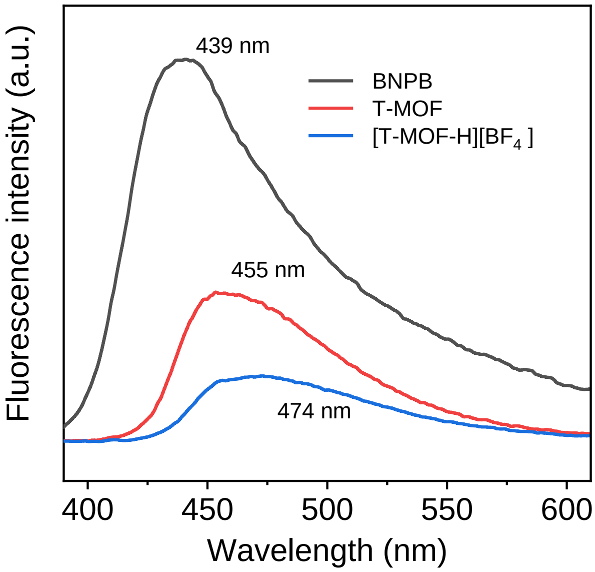

Figure S12. Solid-state fluorescence emission spectra of **BNPB**, **T-MOF**, and **[T-MOF-H]**[BF_4]. Excited at 365 nm.

Table S2. Determination of protonation ratios of **T-MOF** by XPS.

Time	atomic ratio of Zr : B	protonation ratio
0 h	1:0	0 %
2 h	1:0.72	72 %
3 h	1:1	100 %

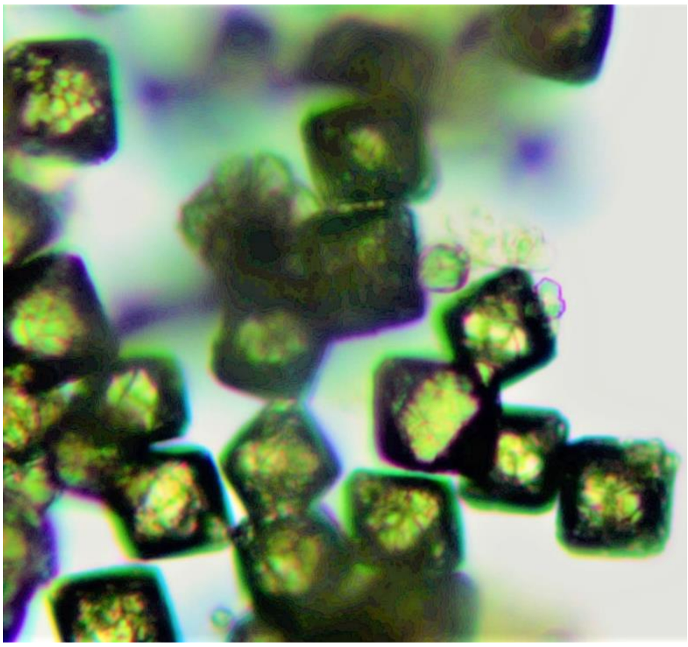

Figure S13. Photograph of **[T-MOF-H]**[BF_4].

7. Assembling pseudorotaxanes inside [T-MOF-H][BF_4]

Freshly prepared [**T-MOF-H**][BF_4] (9.7 mg) was soaked in 1.0 mL of super-dry (water⩽30 ppm) CH_2Cl_2 containing crown ethers (100 mg/mL) for a period (0~72 h) at room temperature. The materials were filtered, washed with CH_2Cl_2, and dried under vacuum overnight. The resulted material was digested in a K_3PO_4 saturated D_2O/DMSO-d_6 (v/v=2:1) solution and subjected to ^1H NMR measurement. The threading ratio of the crown ether to each benzimidazolium moiety was determined from the peak integrations of both ligand and crown ether.

Assembling of 24C8⊂[T-MOF-H][BF_4]:

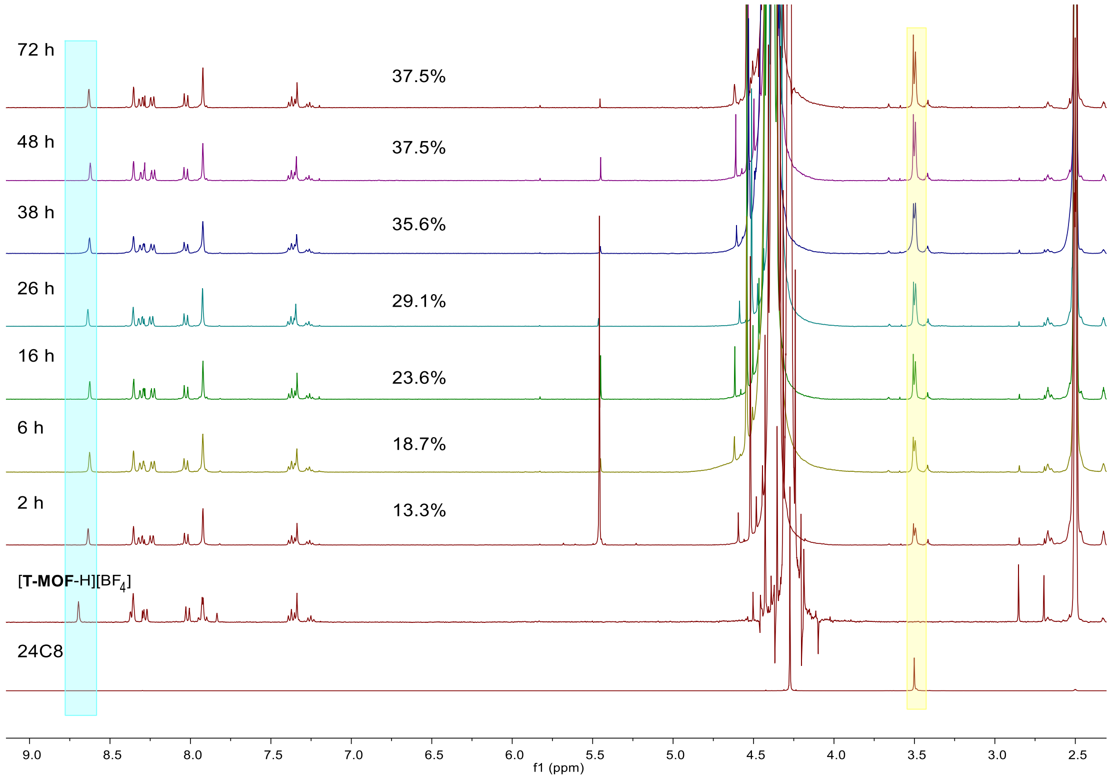

Figure S14. ^1H NMR (400 MHz, 298 K, K_3PO_4 saturated D_2O/DMSO-d_6, v/v=2:1) spectra of digested **24C8**⊂[**T-MOF-H**][BF_4] with variable soaking time. The ratio of **24C8** to ligand are determined based on the integrals of the aromatic proton (8.62 ppm, highlighted in blue) and crown ether protons (3.50 ppm, highlighted in yellow).

Soaking neutral T-MOF in a 24C8-contained solution:

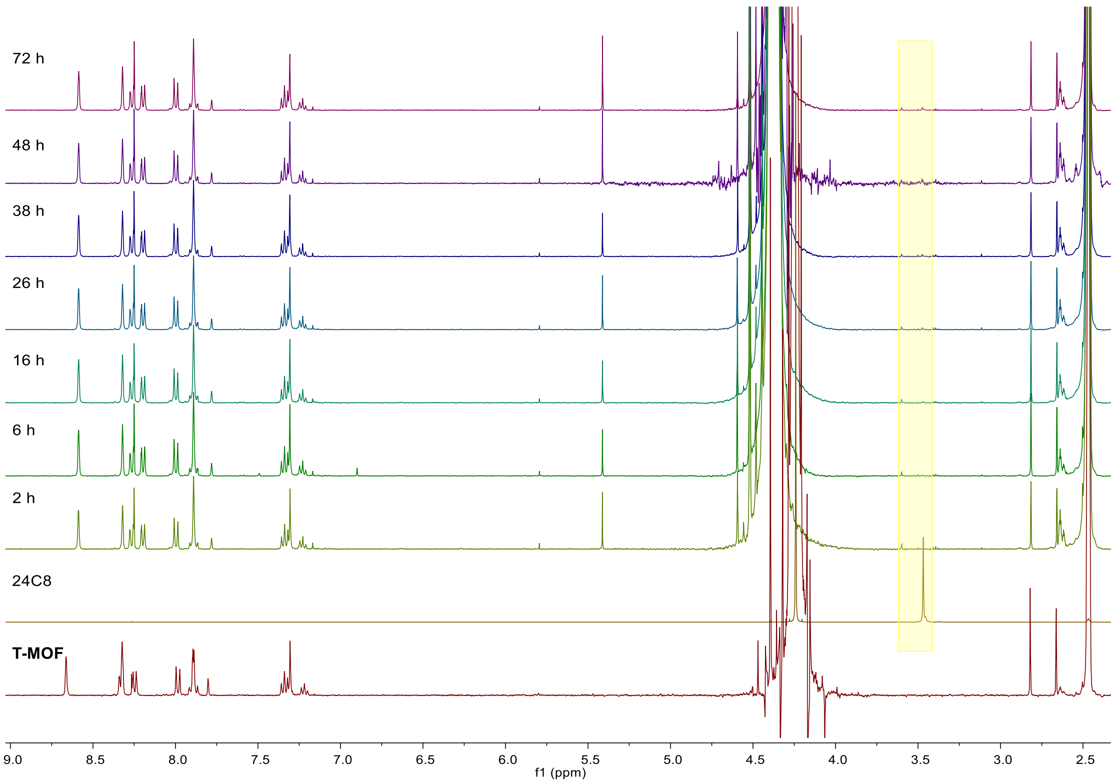

Figure S15. ^1H NMR (400 MHz, 298 K, K_3PO_4 saturated D_2O/DMSO-d_6, v/v=2:1) spectra of digested **T-MOF** obtained with variable soaking time. The crown ether peaks are highlighted in yellow.

Attempt to assemble DB24C8⊂[T-MOF-H][BF$_4$]:

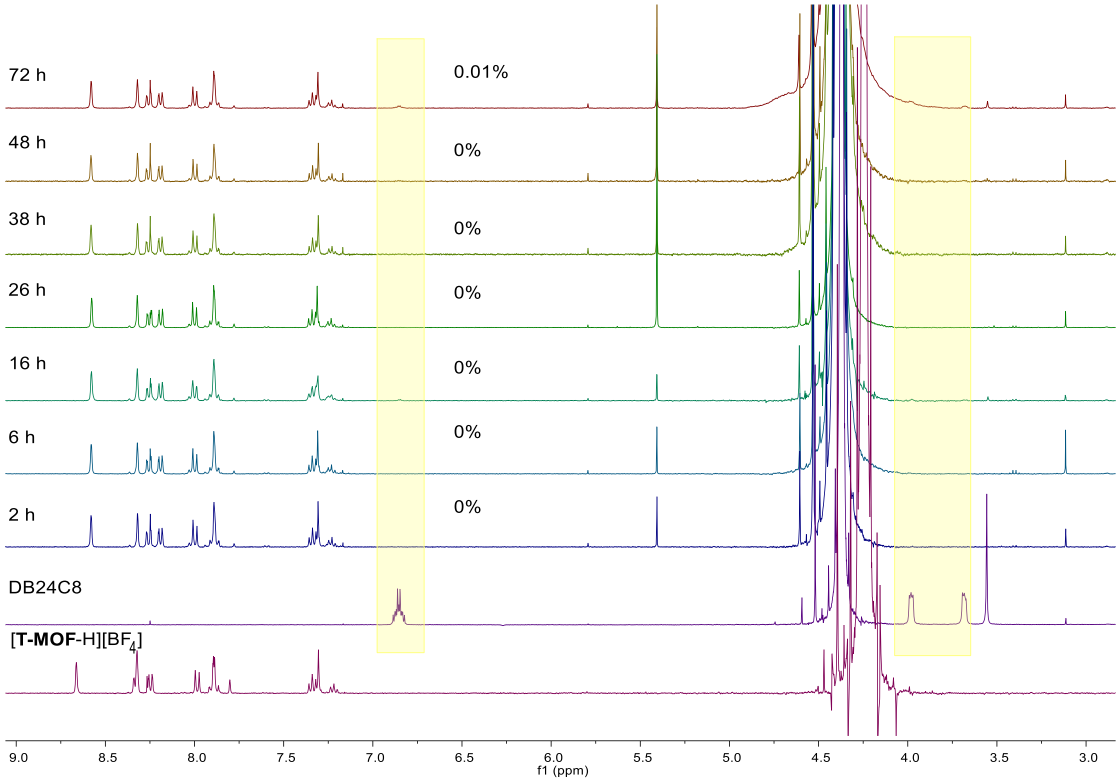

Figure S16. ^{1}H NMR (400 MHz, 298 K, K_3PO_4 saturated D_2O/DMSO-d_6, v/v=2:1) spectra of digested **DB24C8**⊂[**T-MOF-H**][BF$_4$] with variable soaking time. The ratio of **DB24C8** to ligand are determined based on the integrals of the aromatic proton (8.62 ppm) and crown ether protons (6.89 ppm, 4.02 ppm, 3.72 ppm). The crown ether peaks are highlighted in yellow.

Attempt to assemble 18C6⊂[T-MOF-H][BF4]:

(a)

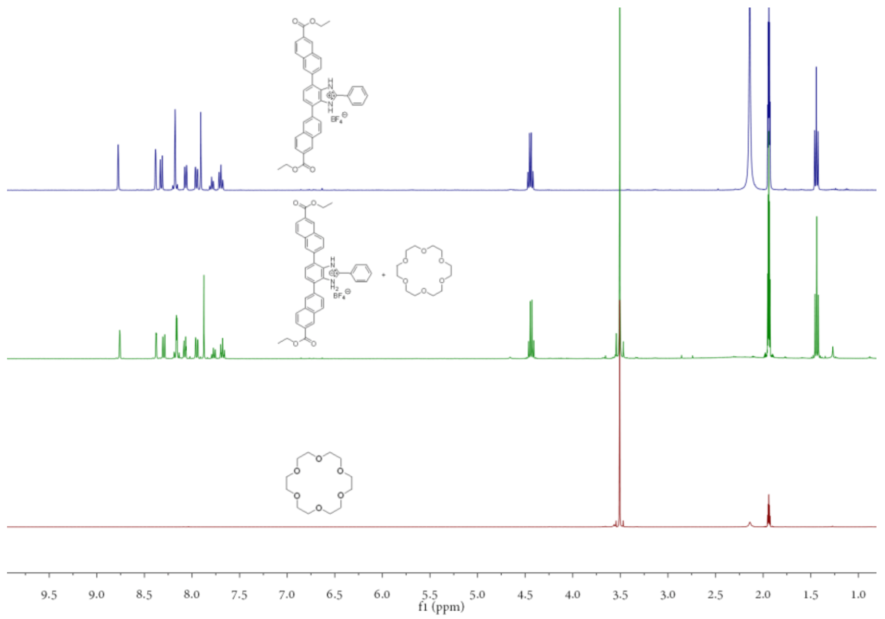

(b)

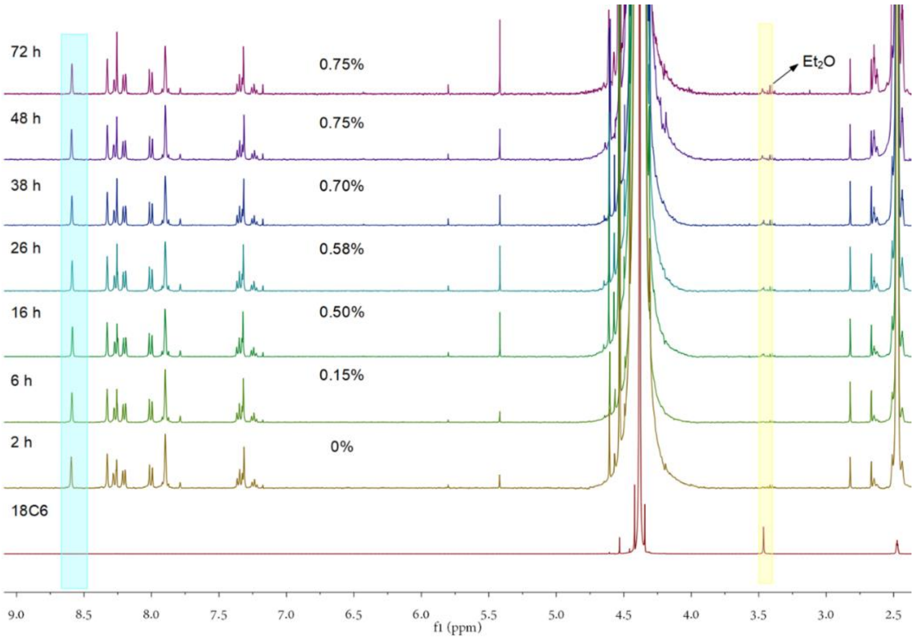

Figure S17. (a) ^{1}H NMR spectra (400 MHz, 298 K, CD_3CN, 4.0 mM) of [**1**-H][BF_4] (top), equimolar mixture of [**1**-H][BF_4] and **18C6** (middle), and **18C6** (bottom). (b) ^{1}H NMR (400 MHz, 298 K, K_3PO_4 saturated D_2O/DMSO-d_6, v/v=2:1) spectra of digested **18C6⊂[T-MOF-H]**[BF_4] with variable soaking time. The ratio of **18C6** to ligand are determined based on the integrals of the aromatic proton (8.62 ppm) and crown ether protons (3.48 ppm). The crown ether peaks are highlighted in yellow.

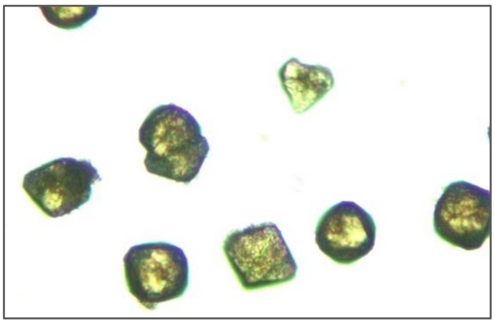

Figure S18. Image of [**T-MOF-H]**[BF_4] soaked in **24C8**-contained solution for 72 hr.

Disassociation of 24C8⊂[T-MOF-H][BF_4]:

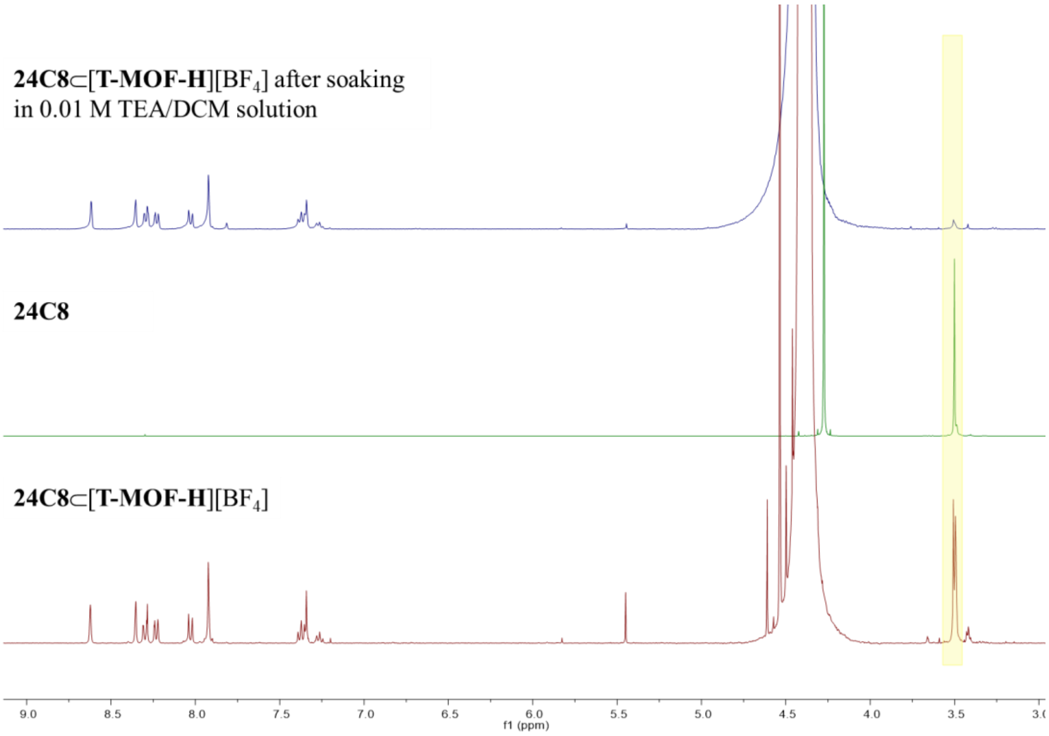

Figure S19. ^{1}H NMR (400 MHz, 298 K, K_3PO_4 saturated D_2O/DMSO-d_6, v/v=2:1) spectra of digested **24C8**⊂[**T-MOF-H**][BF_4], **24C8**, and **24C8**⊂[**T-MOF-H]**[BF_4] after soaking in 0.01 M TEA/DCM solution.

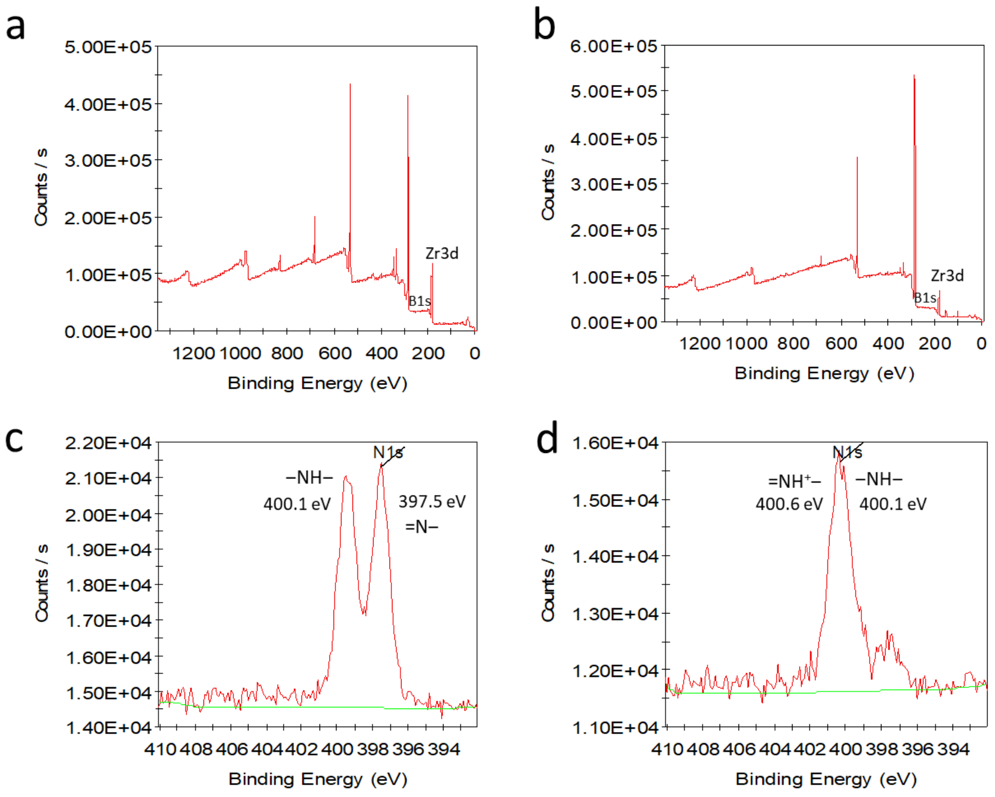

Figure S20. XPS spectra of a) **UiO-66**, b) **UiO-66** treated with HBF_4 solution (0.01 M in CH_2Cl_2) for 3 hr, c) **1**, and d) [**1**-H][BF_4]. The molar ratio of Zr/B for both materials **UiO-66** and HBF_4 treated **UiO-66** was estimated to be 1/24 and 1/24.6, respectively.

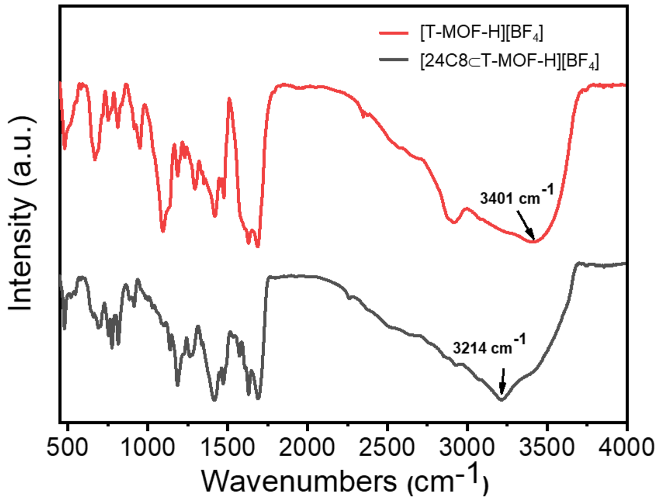

Figure S21. IR spectra of [**T-MOF-H**][BF_4] and **24C8**⊂[**T-MOF-H**][BF_4].

8. Computational modelling and simulated N_2 isotherm

Due to potential sample degradation related to activation, which may block some of the pore entrance for gas adsorption, the N_2 adsorption isotherm measured at 77 K in our experiment showed a relatively small BET surface area of 200 $m^2\ g^{-1}$. To have a better understanding of the porosity of our **T-MOF**, we constructed an idealized structural model (i.e. without linker disorder) of our MOF, optimized the structure at density functional theory (DFT) level, and then we performed Grand-Canonical Monte Carlo (GCMC) simulations to study N_2 adsorption in our MOF. Our predicted N_2 adsorption isotherm at 298 K is shown in **Figure S22**, which shows a nearly linear trend as expected, with the adsorption surpassing that of UiO-66-NH_2 which shows the best N_2 uptake among a series of functionalized UiO-66 MOFs.[S5] This is consistent with the calculated N_2 accessible surface area of 5095 m^2/g, which is higher than that of UiO-68 (with terphenyl dicarboxylate linker) which has a Langmuir surface area of 4170 m^2/g.[S6]

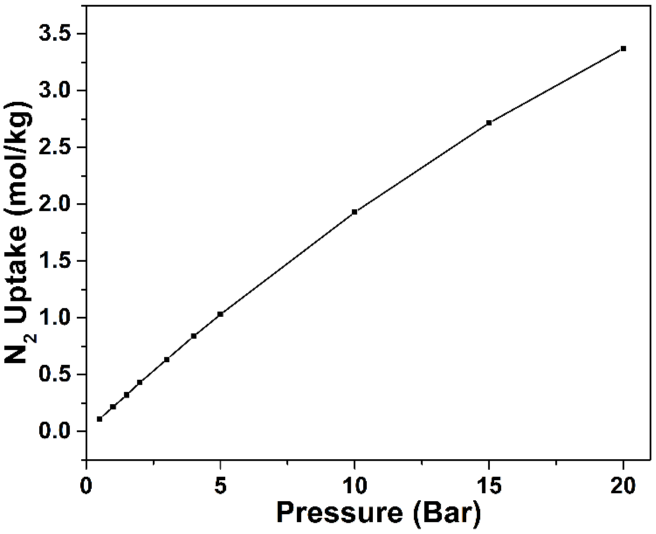

Figure S22. Simulated N_2 adsorption isotherm for **T-MOF** at 298 K.

We also analyzed the pore sizes of **T-MOF**, see **Figure S23** for a histogram of the pore size distributions. As can be found, this MOF features a range of pores with different sizes, with the diameter of the largest pore reaching nearly 14 Å. We also listed relevant pore diameters in **Table S3**.

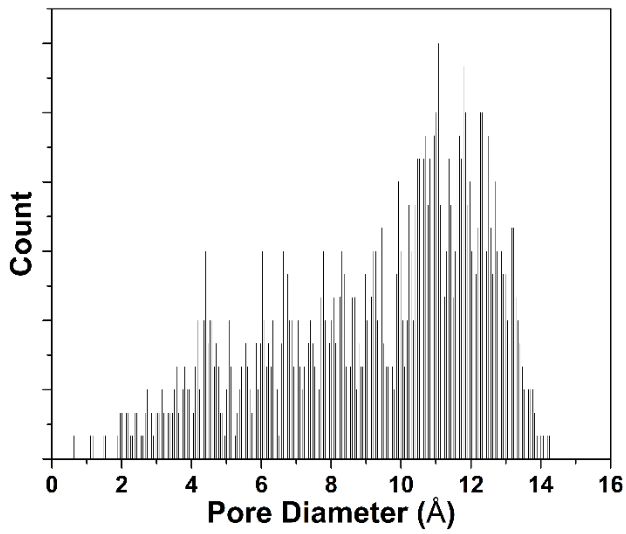

Figure S23: Histogram of pore size distributions.

Table S3: Pore diameters of the simulated **T-MOF**.

Parameter	Value (Å)
diameter of the largest included sphere (Di)	14.7
diameter of the largest free sphere (Df)	6.9
diameter of the largest included sphere along the free sphere path (Dif)	14.7

Computational details: The DFT geometry optimization was performed using the CP2K code (version 8.2), which uses a mixed Gaussian/plane-wave basis set.[S7,S8] We employed double-z polarization quality Gaussian basis sets[S9] and a 400 Ry plane-wave cutoff for the auxiliary grid, in conjunction with the Goedecker-Teter-Hutter pseudopotentials.[S10,S11] Total energy calculations and structural optimizations, including both atomic coordinates and cell parameters, were performed under periodic boundary conditions at the DFT level using the PBE exchange and correlation functional,[S12] with Grimme's D3 van der Waals correction (PBE+D3).[S13] A convergence threshold of 1.0×10^{-6} Hartree was used for the self-consistent field cycle, and structural optimizations were considered to have converged when the maximum force on all atoms falls below 4.5×10^{-4} Hartree/Bohr.

The GCMC simulations and pore size distribution analysis were performed using the RASPA software package.[S14] Cell parameters and positions of framework atoms were taken from our DFT optimized structure, and the partial atomic charges were determined using the REPEAT

method.[S15] The adsorbate-adsorbate and adsorbate-framework interactions are calculated with a pairwise Lennard-Jones potential, where the adsorbate atom ϵ and σ parameters are taken from the TraPPE force field, and the framework atom ϵ and σ parameters are taken from the GenericMOFs force field within the RASPA software package. Individual pairwise interaction parameters are obtained by Lorentz–Berthelot mixing rules.

The geometric characterisation of the porosity, including N_2 accessible surface area and pore diameters, was calculated using the Zeo^{++} software package using high-accuracy settings.[S16]

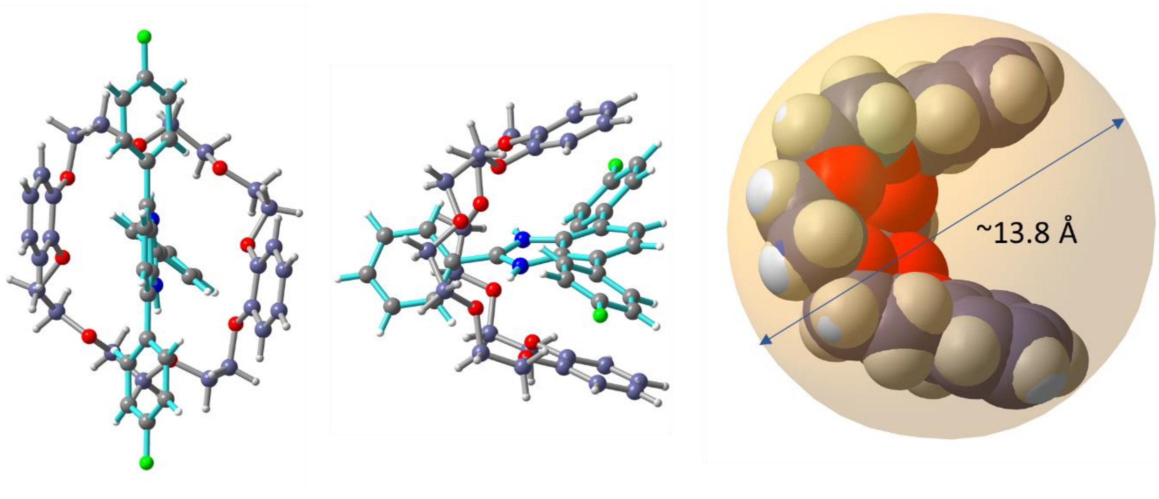

Figure S24: Estimation of the size of crown ether **DB24C8** in a host-guest complex with a T-shaped benzimidazolium axle from the reported crystal structure.[S17] **DB24C8** adopts a C-shaped conformation when forms [2]pseudorotaxanes. The size of the clamped crown ether is estimated by fitting its space-filling model in a sphere.

9. NMR spectra for Compounds

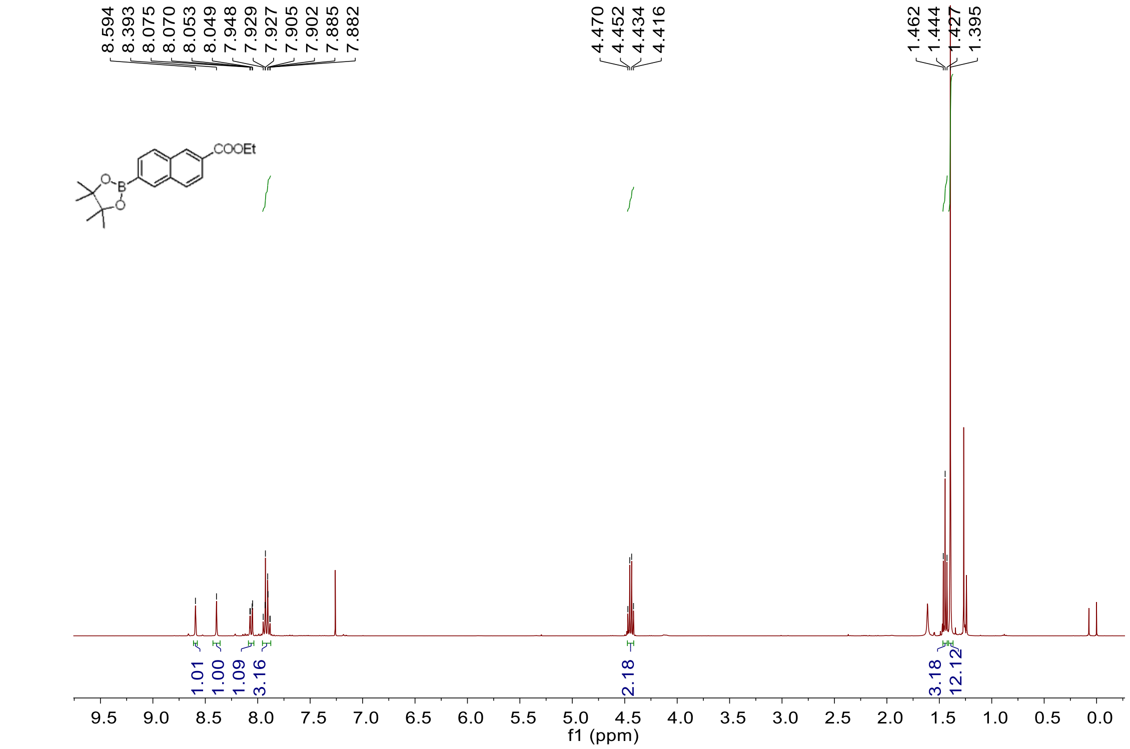

Figure S25. ^{1}H NMR (400 MHz, $CDCl_3$) spectrum of compound **4**.

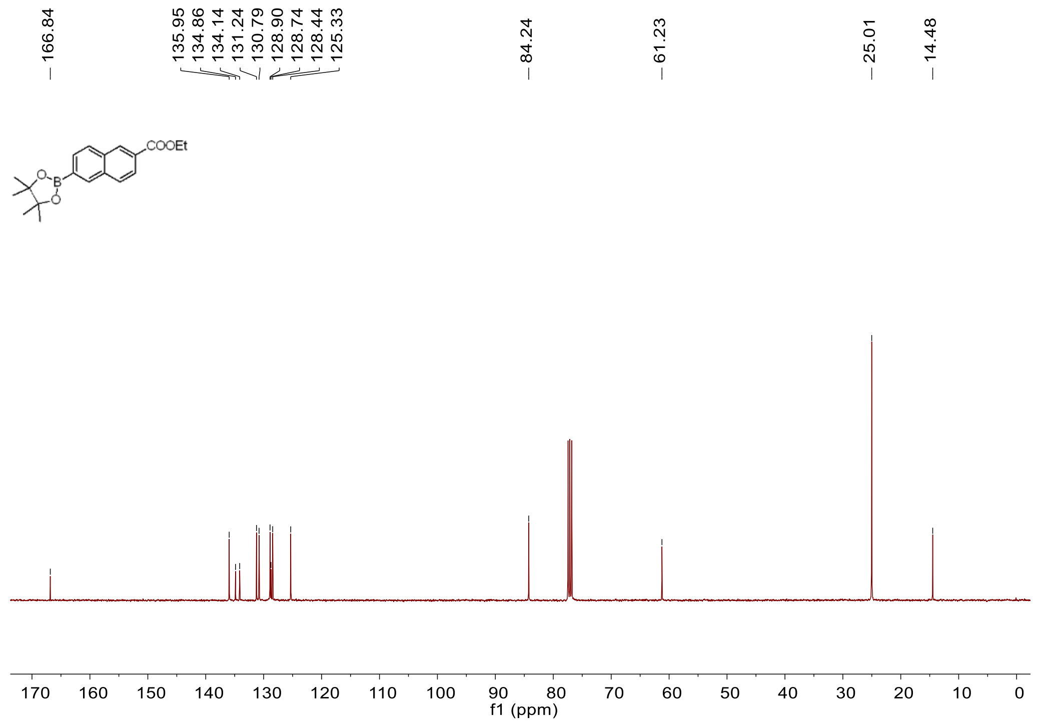

Figure S26. ^{13}C NMR (400 MHz, $CDCl_3$) spectrum of compound **4**.

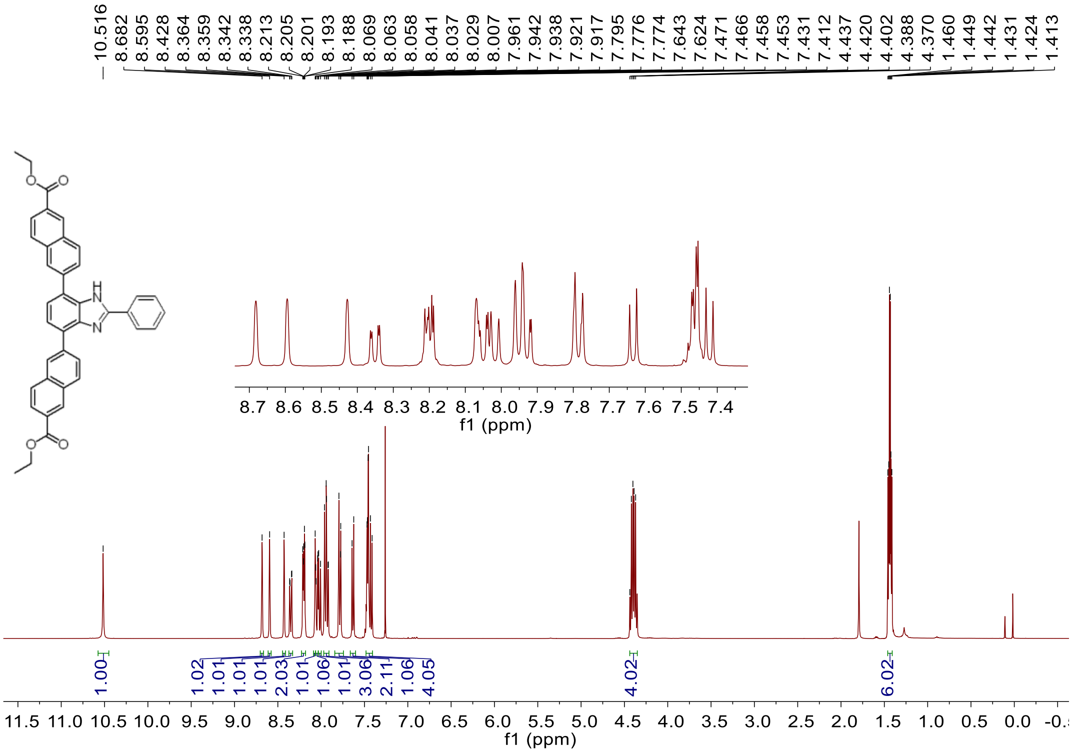

Figure S27. ^{1}H NMR (400 MHz, $CDCl_3$) spectrum of compound **1**.

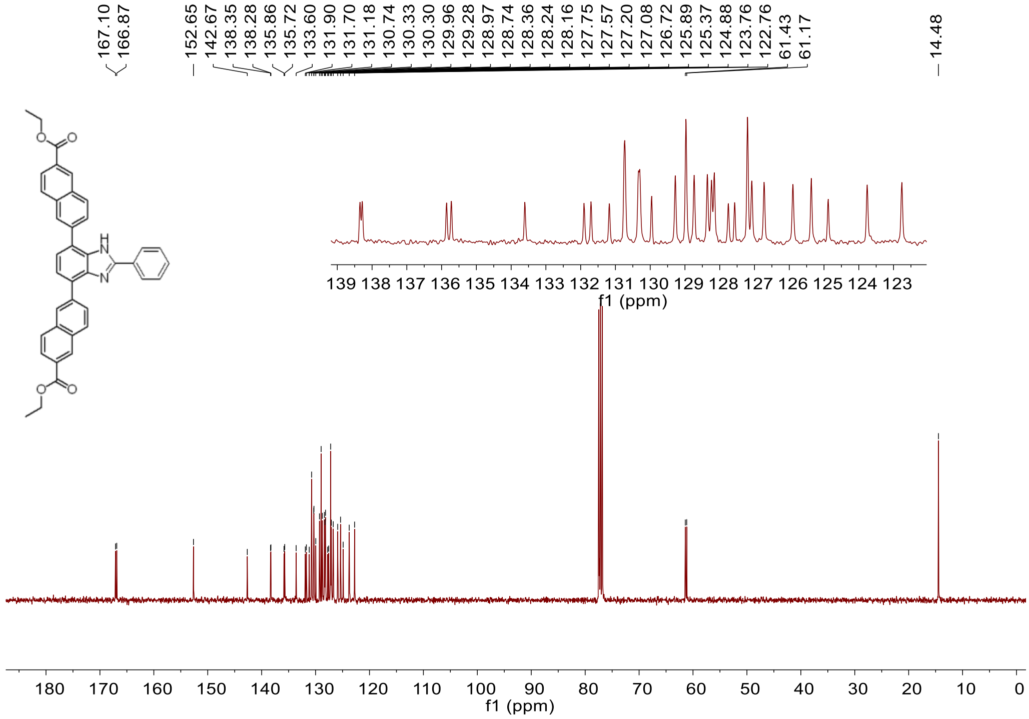

Figure S28. ^{13}C NMR (400 MHz, $CDCl_3$) spectrum of compound **1**.

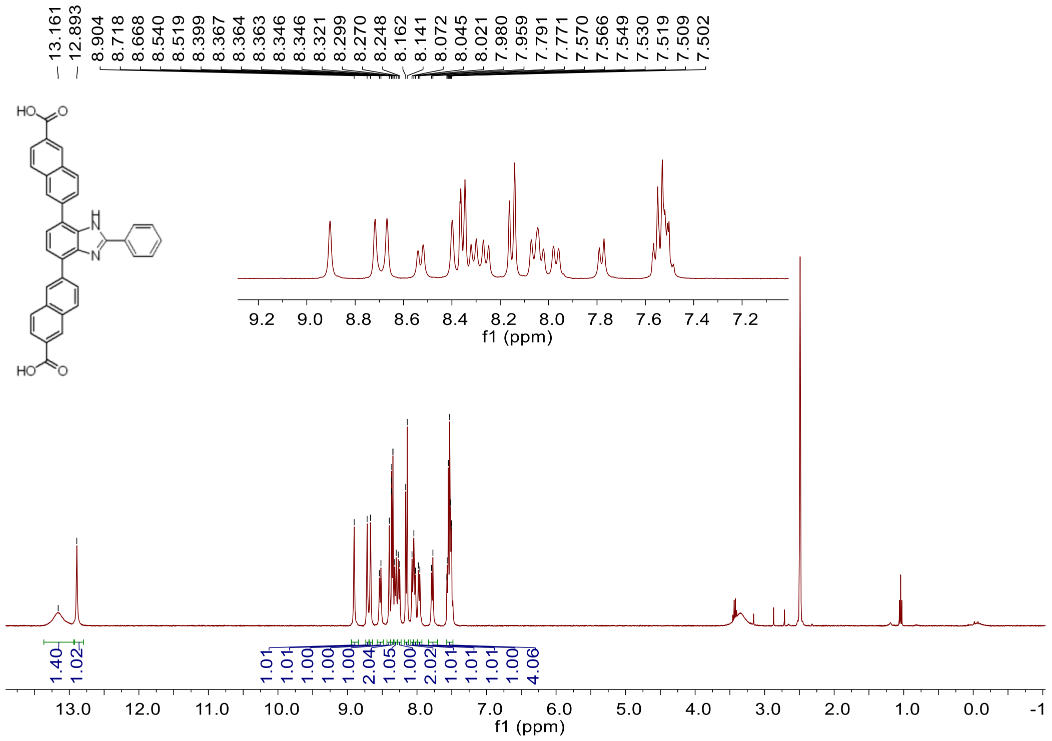

Figure S29. ^{1}H NMR (100 MHz, DMSO-d_6) spectrum of compound **BNPB**.

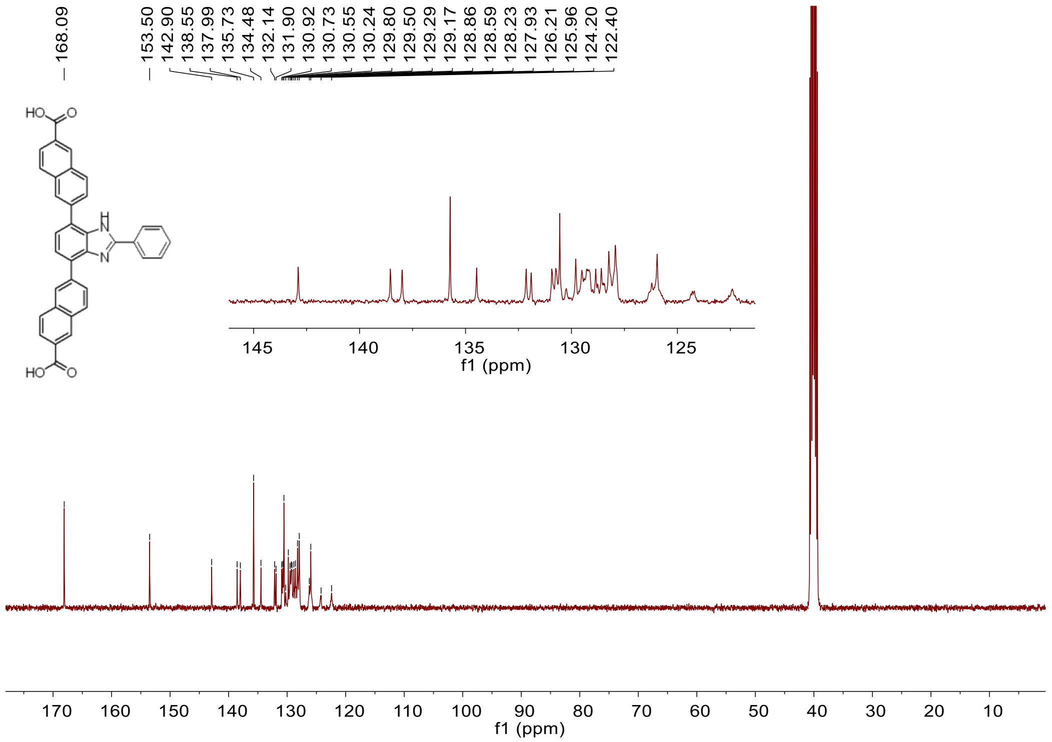

Figure S30. ^{13}C NMR (100 MHz, DMSO-d_6) spectrum of compound **BNPB**.

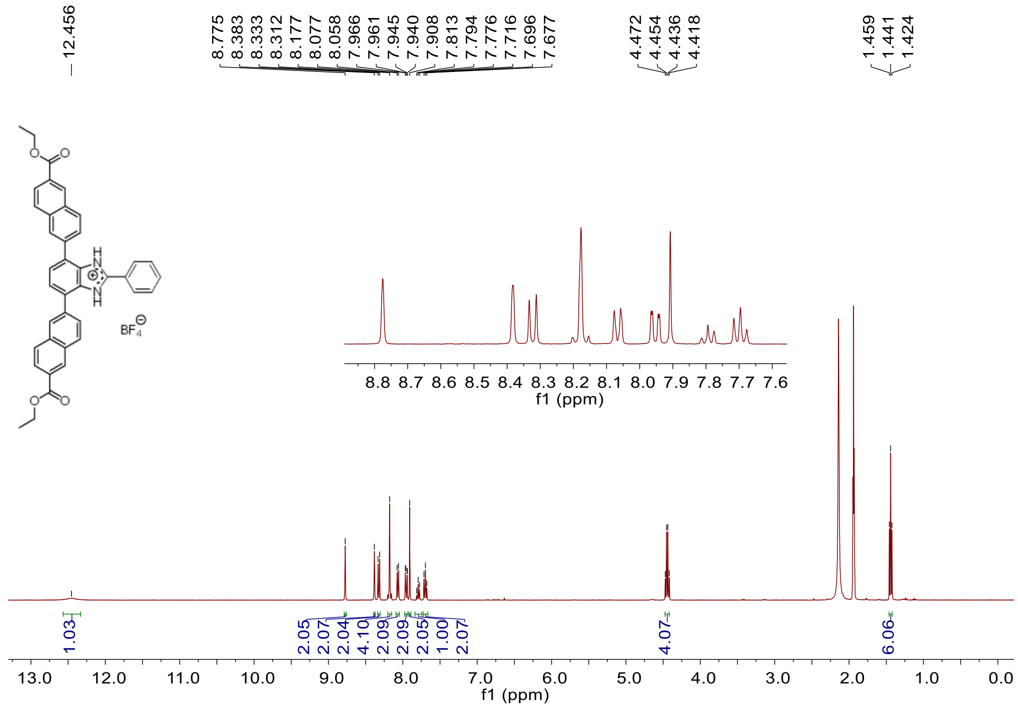

Figure S31. ^{1}H NMR (100 MHz, CD_3CN) spectrum of compound [**1**-H][BF_4].

Reference:

[S1] . N. Noujeim, K. L. Zhu, V. N. Vukotic, S. J. Loeb, *Org. Lett.* **2012**, *14*, 2484-2487.

[S2]. J. T. Joseph, A. M. Sajith, R. C. Ningegowda, S. Shashikanth, *Adv. Synth. Catal.* **2017**, *359*, 419-425.

[S3] O. V. Dolomanov, L. J. Bourhis, R. J. Gildea, J. A. K. Howard, H. Puschmann, *J. Appl. Cryst.* **2009**, *42*, 339-341.

[S4] G. M. Sheldrick, *Acta Cryst. A*, **2008**, *64*, 112-122.

[S5] G. E. Cmarik, M. Kim, S. M. Cohen, K. S. Walton, *Langmuir* **2012**, *28*, 15606-15613.

[S6] J. H. Cavka, S. Jakobsen, U. Olsbye, N. Guillou, C. Lamberti, S. Bordiga, K. P. Lillerud, *J. Am. Chem. Soc.* **2008**, *130*, 13850-13851.

[S7] J. VandeVondele, M. Krack, F. Mohamed, M. Parrinello, T. Chassaing, J. Hutter, *Comput. Phys. Commun.* **2005**, *167*, 103-128.

[S8] J. Hutter, M. Iannuzzi, F. Schiffmann, J. VandeVondele, *Wires. Comput. Mol. Sci.* **2014**, *4*, 15-25.

[S9] J. VandeVondele, J. Hutter, *J. Chem. Phys.* **2007**, *127*, 114105

[S10] S. Goedecker, M. Teter, J. Hutter, *Phys. Rev. B Condens. Matter* **1996**, *54*, 1703-1710.

[S11] M. Krack, *Theor. Chem. Acc.* **2005**, *114*, 145-152.

[S12] J. P. Perdew, K. Burke, M. Ernzerhof, *Phys. Rev. Lett.* **1996**, *77*, 3865-3868.

[S13] S. Grimme, J. Antony, S. Ehrlich, H. Krieg, *J. Chem. Phys.* **2010**, *132*, 154104.

[S14] D. Dubbeldam, S. Calero, D. E. Ellis, R. Q. Snurr, *Mol. Simulat.* **2016**, *42*, 81-101.

[S15] C. Campana, B. Mussard, T. K. Woo, *J. Chem. Theor. Comput.* **2009**, *5*, 2866-2878.

[S16] T. F. Willems, C. Rycroft, M. Kazi, J. C. Meza, M. Haranczyk, *Micropor. Mesopor. Mat.* **2012**, *149*, 134-141.

[S17] K. Zhu, V. N. Vukotic, S. J. Loeb, *Angew. Chem. Int. Ed.* **2012**, *51*, 2168-2172.
